# Identification and Characterization of a Novel Non-Coding RNA Involved in Sperm Maturation

**DOI:** 10.1371/journal.pone.0026053

**Published:** 2011-10-12

**Authors:** Min-Jie Ni, Zhi-Hong Hu, Qiang Liu, Mo-Fang Liu, Min-hua Lu, Jin-Song Zhang, Li Zhang, Yong-Lian Zhang

**Affiliations:** 1 Shanghai Key Laboratory of Molecular Andrology, State Key Laboratory of Molecular Biology, Shanghai, China; 2 Core Facility for Non-Coding RNA, Institute of Biochemistry and Cell Biology, Shanghai Institutes for Biological Sciences, Shanghai, China; 3 Shanghai Institute of Planned Parenthood Research, Shanghai, China; University of Cambridge, United Kingdom

## Abstract

A long and ever-expanding roster of small (∼20–30 nucleotides) RNAs has emerged during the last decade, and most can be subsumed under the three main headings of microRNAs(miRNAs), Piwi-interacting RNAs(piRNAs), and short interferingRNAs(siRNAs). Among the three categories, miRNAs is the most quickly expanded group. The most recent number of identified miRNAs is 16,772 (Sanger miRbase, April 2011). However, there are insufficient publications on their primary forms, and no tissue-specific small RNAs precursors have been reported in the epididymis. Here, we report the identification in rats of an epididymis-specific, chimeric, noncoding RNA that is spliced from two different chromosomes (chromosomes 5 and 19), which we named *HongrES2. HongrES2* is a 1.6 kb mRNA-like precursor that gives rise to a new microRNA-like small RNA (*mil-HongrES2*) in rat epididymis. The generation of *mil-HongrES2* is stimulated during epididymitis. An epididymis-specific carboxylesterase named CES7 had 100% cDNA sequence homology at the 3′end with *HongrES2* and its protein product could be downregulated by *HongrES2* via *mil-HongrES2*. This was confirmed *in vivo* by initiating *mil-HongrES2* over-expression in rats and observing an effect on sperm capacitation.

## Introduction

Cells face a wide range of threats and regulatory demands.Among the many tools available to meet these challenges is a collection of pathways that use small (∼20–30 nucleotides) RNAs to recognize target nucleic acids and present them to specific effector complexes that generally inhibit gene expression [1]. In animals, most of these RNAs could be sorted into three main groups, namely microRNAs (miRNAs), Piwi-interacting RNAs (piRNAs), and short interfering RNAs (siRNAs). Although they share some common features, each RNA category can differ from the others in various ways, including length, precursor structure, cofactor requirement, modification state, sequence bias, and regulatory function, and the differences can themselves vary between species [2].

The recently established and most quickly expanding subgroup of regulatory RNAs, the microRNAs (miRNAs) is composed of 18–25 nucleotide long molecules that control the expression of their target genes via antisense base pairing [3], [4]. The genes encoding miRNAs are initially transcribed as long, primary microRNAs (pri-miRNAs), which vary in length from hundreds to thousands of nucleotides. The pri-miRNAs are then sequentially processed by two RNase-III enzymes, Drosha and Dicer, into a stem-loop pre-miRNA, generating an imperfect double-stranded RNA (dsRNA) duplex that contains both the mature miRNA strand and its complementary strand (miRNA*) [5]. For post-transcriptional gene silencing, the mature miRNA strand is loaded into the effector complex, the RNA-induced silencing complex (RISC), and RISC guides messenger RNAs (mRNAs) to their complementary sequences[6]. Mammalian miRNAs have been shown to be differentially expressed in specific cell types, tissues, and embryonic stem cells. Distinct miRNA expression profiles have also been associated with different diseases and diverse developmental and physiological processes [7], [8], [9].

To avoid designating siRNAs or fragments of other RNAs as miRNAs, miRNAs are identified using a combination of criteria for both their expression and biogenesis. Expression criteria include detection of a distinct ∼22-nt RNA transcript by hybridization to a size-fractionated RNA sample via the northern blotting method (expression criterion A ), and identification of the ∼22-nt sequence in a library of complementary DNAs (cDNAs) made from size-fractionated RNA (expression criterion B). Other criteria include prediction of a potential fold-back precursor structure that contains the ∼22-nt miRNA sequence within one arm of the hairpin.(biogenesis criterion C) [10], phylogenetic conservation of the ∼22-nt miRNA sequence and its predicted fold-back precursor secondary structure (biogenesis criterion D), and detection of increased precursor accumulation in organisms with reduced Dicer function (biogenesis criterion E).

Ideally, a miRNA is identified if an asymmetric ∼22-nt product accumulates (*in vivo*), and is processed from a phylogenetically conserved hairpin precursor by Dicer (A + D + E). However, among these criterion, criterion A is the most important, so that in the absence of processing data, A + D is sufficient. Therefore, a candidate gene can still be annotated as an miRNA gene if they are in the line with .A + C, B + D or D + E [11].

Mammalian spermatogonia undergo mitosis, meiosis, and some morphological changes, becoming fully differentiated, but not mature, sperm in the testis. Most of the sperm maturation occurs in the epididymis [12], [13], so it is an ideal research target organ that could yield better understanding of the molecular mechanism of sperm maturation and provide new ideas for the design of male fertility-control drugs, personalized infertility diagnosis, and treatments and evaluations of sperm health. However, few studies on smRNAs in the epididymis, have been reported [14], [15], [16].

The Mammalian Gene Collection(MGC)and FLJ Human cDNA Database together reported a total of 24,409 cDNAs with full-open reading frames (ORFs) from more than 200 cDNA libraries. This number of genes is close to that predicted by the International Human Genome Sequencing Consortium in 2004 (20,000–25,000). However, an epididymis cDNA library was not present in the sequencing target list.

To identify genes that are important in the rat epididymis, a rat epididymis cDNA library was screened and a 1.3-kb expressed sequence tag (EST ) was found and named *HongrES2* [17]. *HongrES2* was subsequently identified as a novel, 1.6-kb, epididymis-specific, mRNA-like, chimeric noncoding RNA that produces a microRNA like small RNA (*mil-HongrES2*). *Mil-HongrES2* down-regulates CES7 gene expression and is involved in the sperm epididymal maturation process.

Inter-chromosomal chimeric RNAs have been reported in a variety of organisms. These encode proteins [18], [19] that are present in abnormal cells, such as cancer cells. Sklar's group reported a neoplastic gene fusion that mimics the trans-splicing of RNAs in normal human endometrial stromal cells [19], [20]. Recently, a genome-wide screening of chimeric RNAs in budding yeast, fruit fly, mouse, and human genomes identified thousands of chimeric transcripts in all organisms except for yeast, in which only five chimeric RNAs were observed [21]. To the best of our knowledge, the 1.6-kb chimeric transcript *(HongrES2)* identified in the present study is the first reported epididymis-specific full-length non-coding RNA derived from two chromosomes.

## Materials and Methods

### Animals

Healthy male Sprague Dawley (SD) rats were purchased from the Animal Center of the Chinese Academy of Sciences (Shanghai, China). They were housed for 7–10 days in the animal housing at our institute before manipulation. Food and water were freely available throughout the experiments. The protocol conforms to internationally accepted guidelines for the humane care and use of laboratory animals. All research involving animals were conducted according to the approval of the Institute Animal Care Committee of Shanghai Institute of Biochemistry and Cell Biology. The approved permit number for this study is “SYXK2007-0017”.

### Total RNA and small RNA isolation and northern blot analysis

Tissue samples were obtained from male rats after they were sacrificed, and the samples were immediately frozen in liquid nitrogen. Total RNA was extracted for northern blot analysis, which was performed according to a previously described procedure [22]. Twenty micrograms of total RNA from each sample was loaded into each lane. The probe was a ^32^P-labeled 504-bp cDNA fragment (seqNo 861-1364-nt) of rat *HongrES2* that was cut from the vector (T-easy-H2). An 18S rRNA hybridization signal was used as a loading control.

The Ambion mirVana^TM^ miRNA Isolation Kit was used for small RNA isolation, according to the manufacturer's protocol. Small RNAs (30 µg per sample, <200 bp), including miRNAs, were separated on a 12% acrylamide/8M urea denaturing polyacrylamide gel before being transferred to an Ambion BrightStar–Plus Nylon membrane.

Hybridization was carried out using a 24 bp LNA probe purchased from Exiqon (Woburn, MA, USA). Northern blot analysis was performed according to a protocol published on the Exiqon website (http://www.exiqon.com). All of the Northern blot analyses were carried out at 65°C under high stringency conditions.

### RACE analysis, PCR performance ,ORF identification and secondary structure analysis

**Figure 1:**
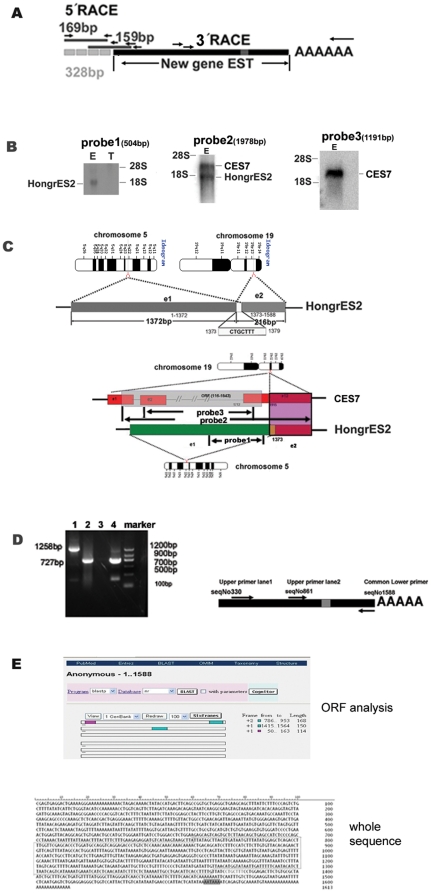
Clone and sequence analysis of *HongrES2* cDNA. (A) Schematic representation of the 1.3-kb EST screened from a rat epididymal cDNA library, and the new 328 bp sequence obtained from two round 5′ RACE by a BD and Ambion kit, respectively. (**B**) Left panel: Northern blot analysis for *HongrES2* with ^32^P-labeled 504-bp *HongrES2* EST probe1 (seqNo861-1364) . E: epididymis; T: testis; 20 µg total RNA per lane; middle panel: Northern blot analysis for *HongrES2* with ^32^P-labeled 1978 bp *CES7* EST probe2 (seqNo116-2094); right panel: Northern blot analysis for *HongrES2* with ^32^P-labeled 1191 bp *CES7* EST probe3 (seqNo498-1689). (**C**) Upper panel: Chromosomal localization of the *HongrES2* gene. The dark gray box is exon 1 from rat chromosome 5, and the light gray box is exon 2 from chromosome 19. The small white box represents the 7-nucleotide overlap from these two exons. e1, exon1; e2, exon2. Lower panel: A sequence alignment of CES7 and *HongrES2*. Twelve exons of the CES7 cDNA are depicted in the red rectangle. The same 216- bp fragment of the 3′ end of CES7 exon 12 and *HongrES2* exon 2 is depicted by a purple shadow. The location of probe1, probe2 and probe3 for Northern blot analysis in figure1B is also marked out. (**D**) Left panel: RT-PCR validation using primers spanning the chimeric junction portion of HongrES2 cDNA. The product in lane1was got by the primer pairs of upper primer lane1 and common lower primer showed on the right panel. The product in lane2 was got by the primer pairs of upper primer lane2 and common lower primer; right panel: the schematic representation of the location of the two primer pairs used in the RT-PCR. Lane3: negative control; lane4: positive control. (E) Upper panel: ORF analysis of HongrES2 cDNA on line. Three short frames were displayed by rectangles and their sequential number and length were listed. Low panel: The full-length gene sequence of the *HongrES2* cDNA. The gray box represents the poly(A) addition signal. The line labels the probe1 sequence used for the northern blot and *in situ* hybridization in Figure 3A.

**Figure 2:**
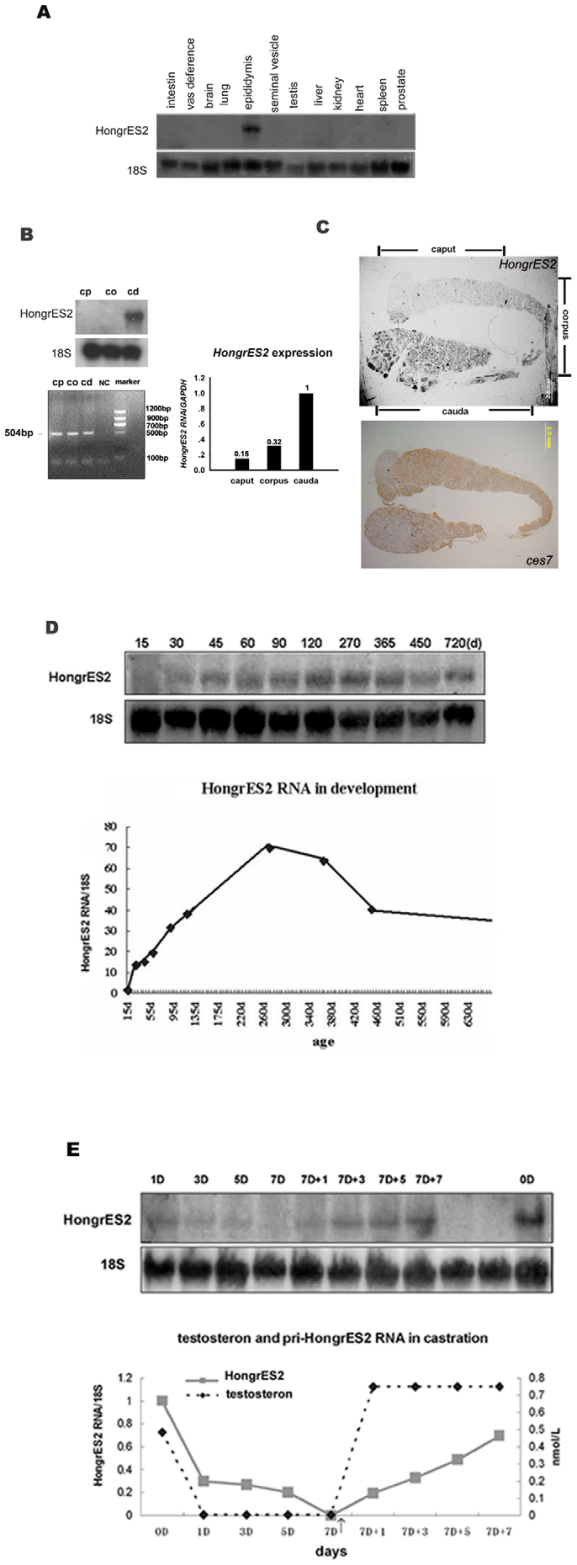
Regional and temporal expression of *HongrES2* RNA. (**A**) The tissue distribution of *HongrES2* RNA. (**B, C**) The region-specific expression pattern, as shown by northern blot analysis, QRT-PCR ,and *in situ* hybridization. CP:caput; CO:corpus; CD: cauda; an 18S probe was used on the stripped membrane as an internal loading control. Bar = 2.0 mm. The primers used for RT-PCR ,Real-time PCR was listed in Table1. (**D**) The upper panel shows the northern blot analysis of *HongrES2* RNA and 18S rRNA during development. The lower panel shows the relative amounts of *HongrES2* RNA in the rat epididymis at different developmental stages. (**E**) The upper panel shows Northern blot analysis of adult rat epididymal *HongrES2* RNA. level from pre-castration (d0) and castration for 1, 3, 5, and 7 days (d1, d3, d5, and d7) as well as for 1, 3, 5, and 7 days after the initial injection of testosterone propionate applied to the 7d-castrated rats (d7+1, d7+3, d7 +5, and d7 +7). Injections were continued every 2 days. The lower panel showed the relative expression levels of *HongrES2* RNA (hybridization density of *HongrES2* RNA/18S ribosomal RNA) in the rat.

The BD SMART™ RACE cDNA Amplification and the Ambion First Choice RLM-RACE kits were employed for first and second rounds of 5′RACE, respectively, according to the manufacturers' protocols, two arounds of PCR amlifications were used, and 40 cycles of amplification were used in each round. Amplification of the 3′ end (3′ RACE) was performed by the Ambion FirstChoice RLM-RACE kit. The Amplified fragments were cloned into pGEM-T-Easy (Promega) for sequence analysis. RT-PCR for the 1258 bp fragment of *HongrES2* cDNA in Figure 1D was amplified with nested PCR strategy (two arounds with different upper primer and the same lower primer). The 727-bp and 504-bp fragments of *HongrES2* cDNA in Figure 1D and Figure 2B was gained without nested PCR. The Takara Ex -Taq PCR reaction system was applied, the annealing temperature was 55°C, and the amplification was carried out under 40 cycles.

ORF searching of the full-length sequence of *HongrES2* was carried out using the ORF Finder Program (http://www.ncbi.nlm.nih.gov/gorf/gorf.html). The secondary structure of the sequence, beginning from seqNo 1365 and extending to seqNo 1588, was predicted by the mfold server (http://mfold.rna.albany.edu/?q=mfold/download-mfold).

### Real-Time PCR

For mRNA quantification,a total of 2 ug of RNA prepared as described above was used for reverse transcription, which was performed with a ReverTra Ace-α-TM kit (Toyobo Co., Osaka, Japan) according to the manufacturer's instructions. Real-time PCR was performed in a total volume of 20 ul of reaction mixture containing 10 ul of SYBR Green Real-time PCR Master Mix (Toyobo).Amplification of gene of interest cDNA was normalized to that of Gapdh. Results are calculated by two standard curve methods and expressed as the fold-increase of gene of interest cDNA compared with the control. For small RNA quantification an improved methods were carried out according to the previous publication [23].

The gene of interet (*mil-HongrES2*)was defined as 1 U,and the other was normalized to it

### Tissue section preparation and in situ hybridization

Adult rat tissue slides were prepared as described previously [22]. *In situ* hybridization was carried out according to previously described methods [24]. DIG-labeled antisense and sense RNA probes were transcribed by T7 and SP6 RNA polymerase (Promega) from the pGEM T-easy-H2 vector and the pGEM-T-easy-bin1b vector, and NBT/BICP (Roche) was used as the AP substrate to generate a purple signal. The FITC-labeled antisense probe was transcribed by SP6 RNA polymerase from the pGEM-T-easy-CES7 vector, and INT/BCIP (Roche) was used as the AP substrate to give a brown signal.

### Transfection and luciferase assay

Transfections were performed using Lipofectamine 2000 (Invitrogen), according to the manufacturer's protocol. For examination of the reduction of CES7 protein expression, the pcmv-tag4a-CES7 and Pcmv-tag4a-H2 (pcmv-tag4a-H2T or pcmv-tag4a/mock) constructs (1∶3.5) were co-transfected into PC1 cells [25] cultured in 6-well plates. For examination of Renilla luciferase activity variation, pRL-TK vectors were constructed with the 3′ end of CES7, pGL-3 vectors were used as an internal control and the *HongrES2* expression vector (1∶0.1∶2) was co-transfected into cells cultured in 96-well plates. The relative luciferase activity was assayed by Berthold multilabel reader Mithras LB940 after 48 hours of culture. The vector for the analysis of luciferase activity was pRL-TK (Promega), with the wild-type 271 bp fragment of the CES7 3′ end (seqNo1858-2129) sequence or its target site mutant 3′ end fragment obtained by RT-PCR.

### Protein extracts and Western blot analysis

Total protein extracts of the cultured cells after transfection were prepared using RIPA buffer with protease inhibitors. Total protein extracts for each cell sample (30 µg/lane) were separated onto 12% SDS-PAGE gels and transferred to polyvinylidene difluoride membranes (Amersham Pharmacia Biotech). A polyclonal antiserum against the recombinant rat CES7 protein was used as the primary antibody (dilution 1∶10,000). The secondary antibody was a goat horseradish peroxidase (HRP)-conjugated anti-rabbit immunoglobulin G (IgG; dilution 1∶20,000; CalBiochem). Peroxidase activity was detected with a chemiluminescence substrate (Western Blot Chemiluminescence Reagent Plus; Amersham Pharmacia Biotech). The vector used for the expression of the *HongrES2* RNA and CES7 protein in PC1 cells contained the full-length sequence (pcmv-tag4a). A mouse monoclonal antibody specific for Dicer (cat. No. ab14601) was purchased from Abcam and used at a 1∶500 dilution. A polyclonal antibody against FLAG was purchased from Sigma (F7425) and used at a 1∶300 dilution. All quantifications were carried out using Labworks software.

### Immunoprecipitation

PC1 cells were co-transfected with the *HongrES2* expression plasmid and a FLAG-tagged hAgo2 expression plasmid (a gift from Li-Gang Wu, SIBS) using Lipofectamine 2000 (Invitrogen). Forty-eight hours after transfection, cells were collected by scraping and resuspended in 500 µL lysis buffer (50 mM Tris [pH 7.5], 150 mM NaCl, 2 mM MgCl_2_, 2 mM CaCl_2_, 0.5% Nonidet P-40, and 1 mM dithiothreitol). Lysates were cleared by centrifugation at 16,000× g for 10 minutes. One third of each cleared lysate was added to 1 mL Trizol and saved for assaying the total input. The remaining lysates were mixed with 15 µl anti-FLAG rabbit polyclonal antibody-conjugated M2 beads (Sigma cat.# F7425) and rotated at 4°C overnight. The beads were then sedimented by centrifugation and washed 3 times in lysis buffer. Eighty percent of the beads were resuspended in 1 mL Trizol for RNA isolation, and 20% of the beads were resuspended in 20 µL 2X complete Laemmli buffer for protein analysis [26].

### siRNA interference

Two siRNA sequences targeting the ORF of the mouse Dicer1 gene were designed using published sequences [27], [28]. The siRNAs were transfected using Lipofectamine 2000 at a level of 6 µg siRNA per dish (100-mm dish). The cells were collected after 48 hours for subsequent analysis.

### Infection SD rat model construction

Pathogenic bacteria were obtained from human patients with epididymitis and 3 adult SD rats were inoculated with cultured bacteria in their cauda tubules. NaCl solution (0.9%) was used as a negative control and was injected into the cauda tubules in 3 additional adult SD rats. The volume injected per animal was 25 µL, and the amount had an optical density of A_OD600_ = 3.0. The Staphylococcus and Morganella strains were cultured about eight hours and their optical density was examined and adjusted equally to A_OD600_ = 1.0 (1OD bacteria = 2.5×108 cfu/ml)by the bacteria culture solution(LB solution) before they were injected into the rat epididymis. The scrotum was not cut open when the rats were injected. The treated animals were fed for 4 days before being sacrificed. The pathogenic bacteria of human patients with epididymitis were obtained from Shanghai Jiao Tong University School of Medicine. Informed consent was obtained from all participants, this consent was written, and the study was approved by the Institutional Ethics Board of School of Medicine, Shanghai Jiao Tong University.

### Construction of the small RNA library

Rat epididymis microRNA libraries were constructed to find novel microRNAs expressed in this male organ, and the caput sub-library was used. The basic protocol is described in the “Cloning of Small RNA Molecules” published by *Current Protocols in Molecular Biology* (2003) 26.4.1-26.4.8. The total RNA of each tissue was extracted using the Trizol reagent (Invitrogen). The fraction of 18–25 nt small RNAs was excised from a 12% acrylamide/8 M urea denaturing polyacrylamide gel and purified to construct the small RNA library. Adapters for the 3′ and 5′ ends were ligated onto the RNA, and the following steps were carried out according to the method of “Cloning of Small RNA Molecules” of *Current Protocols in Molecular Biology* [29], [30].

### Mil-HongrES2 over-expression SD rat model construction

An miRNA analog (agomir) of *mil-HongrES2* was purchased from GuangZhou RiboBio.Co., Ltd [31], and injected into the cauda epididymis of adult male rats (450 g–500 g) at a dose of 4 nmol per side. A scrambled miRNA agomir was used as the negative control. The animals were sacrificed 3 days after injection for further analysis.

### Evaluation of sperm capacitation

We applied the protocol of protein tyrosine phosphorylation assessment as used in previous studies. The spermatozoa from the cauda epididymis of the *mil-HongrES2* over-expression rats and the control rats were released into the capacitation medium (94.6 mM NaCl, 25 mM KCl, 1.71 mM CaCl_2_, 1.19 mM MgSO_4_, 1.19 mM KH_2_PO_4_, 25 mM NaHCO_3_, 5.56 mM glucose, 10.76 mM sodium lactate, 0.5 mM sodium pyruvate, 0.002% phenol red, 4 mg/ml bovine serum albumin; 50 mg/ml streptomycin sulfate, and 75 mg/ml potassium penicillin, pH 7.4, osmolarity ∼310 mosmol/kg). The sperm pellet was suspended in SDS sample buffer, and total sperm protein was separated by SDS-PAGE with 8% Tris-glycine gels. Tyrosine-phosphorylated proteins from the spermatozoa were detected by western blotting with mouse monoclonal anti-phosphotyrosine4G@ antibody(Millipore,catNo 05-321) at a diluton of 1∶10,000, and the α-tubulin was used as the internal control.

### Remarks

The sequences of primers, probes, and siRNAs used are listed in Table S1. The vectors used are listed in Table S2.

## Results

### Cloning of a novel mRNA-like transcript (*HongrES2*)

To identify the homologous gene of monkey CES7 in rat, a rat epididymis cDNA was screened , using a 163 bp probe of a mouse sequence that was homologous to the 3′ end fragment of the monkey CES7 gene ( the probe sequence see Figure S1). Among the plaques we obtained, except for the rat homologous CES7 gene EST, which has already benn reported [32], [33], another two new gene ESTs were found as by-products and named *HongrES1* EST and *HongrES2* EST. Over the past several years our lab has sequentially reported the identification and biological function of the new gene *HongrES1* which was a new member of serpin family in rat epididymis [17] [34].

The present paper focused on the identification and characterization of the remaining 1.3 kb new gene *HongrES*2 EST (Figure 1A). Northern blot analysis with a region of this *HongrES2* EST (504-bp) as a probe (probe1 in Figure1C, lower panel) detected an mRNA (approximately 1.6 kb) in the rat epididymis (Figure. 1B, left panel).

Two additional cDNA fragments, 159-bp and 169-bp in size, were obtained after two rounds of 5′RACE. No additional sequences were obtained with a third round of 5′RACE. The newly achieved 328-bp cDNA by 5′ RACE was continued on genome and combined with the 328 bp additional cDNA fragment, the full-length *HongrES2* cDNA was 1588-bp, which was roughly equal in size to the mRNA detected in the northern blot analysis.

The ORF Finder Program was used to identify the potential ORF in *HongrES2* cDNA. Unexpectedly, many stop codons were spread uniformly throughout the sequences and no ideal ORFs could be found. Only three short fragments were spread along the entire transcripts with average length of about 144-bp (Figure. 1E, upper panel). An added poly(A) signal (AATAAA) was found at the 3′ end of *HongrES2* cDNA, because *HongrES2* was screened from a cDNA library constructed with an oligo dT primer(Figure. 1E, lower panel). Using an Ambion First Choice RLM-RACE kit, which can be used to identify full-length mRNAs with a 5′ cap, the 5′ terminal sequence of *HongrES2* cDNA was successfully obtained. Thus, *HongrES2* was found to be an mRNA-like, non-coding RNA with a 5′ cap and 3′ poly(A) tail.

With further genomic sequence analyses, this transcript was unexpectedly found to be chimeirc, that is derived from two different chromosomes: chromosome 5 for 1–1379 nt of the cDNA, and chromosome 19 for 1373–1588 nt of the cDNA. The two chromosome shared seven nucleotides (CTGCTTT) , which may mark a potential splicing site. The genome location of the 5′ fragment for 1–1379 nt of the *HongrES2* cDNA was in the chromosomal region 5q22–23 and the 3′fragment for 1373–1588 nt in the region 19p13–14 (Figure. 1C, upper panel). Using Blast analysis and comparing the sequences of the *HongrES2* and rat *CES7* cDNA carefully, the two genes were found to share one common 3′ end, which is a 216-bp cDNA fragment from chromosome 19 (Figure. 1C lower pannel). The fragments on each chromosome in this chimeric transcript were contiguous without introns. Using the Ambion FirstChoice RLM-RACE kit, the *HongrES2* 3′ end sequence was validated by 3′RACE spanning two sides of the junction portion, which suggests that the 5′ and 3′ ends of *HongrES2* were in the same transcript (Figure 1A) and ensuring that this was not a cloning artifact. Meanwhile, reverse transcription polymerase chain reation (RT-PCR) of rat cauda epididymis RNA and two different primer pairs spanning the junction portion was carried out to check the corresponding chimeric transcripts (Figure. 1D). The 1258-bp band in lane1 was cloned and sequenced to confirm that it was exactly the *HongrES2* sequence. Northern blot analysis was performed with another probe (probe2 in Figure. 1C, lower panel) containing the 3′end region (from chromosome 19 ) of this *HongrES2* EST to further check that the cloned cDNA of *HongrES2* contains sequences from two separate chromosomes and were not generated because of an artifact during PCR. Two bands were detected(Figure 1B middle panel). The lower band has the same size as the band obtained by probe1 in rat epididymis and the higher band (approximately 2 kb) was set as the signal of rat *CES7* mRNA, because when the rat *CES7* specific probe (probe3 in Figure. 1C lower panel) containing only the 5′end region of *CES7* cDNA was used only one band of approximately 2-kb was detected in rat epididymis (Figure 1B right panel).

These results showed that *HongrES2* was a mRNA-like chimeric noncoding RNA. Its full-length cDNA sequence was submitted to GenBank (Accession No FJ201982) and was presented in Figure 1E, lower panel.

### *HongrES2* RNA is expressed spatially and temporally in the epididymis

According to Northern blot analysis, *HongrES2* RNA was predominantly expressed in the adult epididymis and was not detected in eleven other tissues tested (Figure 2A). *HongrES2* RNA was detected in the cauda region of the epididymis when probe1 was used in the Northern blot analysis of rat epididymis total RNA (Figure 2B, upper panel). However, *HongrES2* transcripts could be detected in both the caput and corpus region, under the detection sensitivity of RT-PCR.. Real-time PCR analysis revealed that *HongrES2* RNA was predominantly expressed in the cauda region, about 70% and 85% amount off in the corpus and caput regions respectively.(Figure 2B, lower panel). These implied that although *HongrES2* RNA was expressed in the caput, corpus, and cauda regions of rat epididymis, its expression level and/or degradation rate (RNA stability) might not be uniform throughout the epididymis tubules, which was exhibiting ” spatial proneness”. *In situ* hybridization of *HongrES2* revealed that the signal was mainly localized in the distal cauda region,a weak signal in the initial segment of the caput epididymis was detected with longer staining time.*In situ* hybridization of CES7 indicated intense signals throughout the whole rat epididymis from distal caput to proximal cauda region,which was quite differet from the expression pattern of *HongrES2* (Figure. 2C).

Northern blot analysis was performed to determine the onset of *HongrES2* RNA expression. *HongrES2* RNA began to be detected approximately at 30 days of age. It increased gradually and remained at a stable level until the animal was 450 days age, when the expression level was reduced slightly (Figure. 2D). The expression was also up-regulated by androgen, which was determined in castrated animals (Figure. 2E).

### *HongrES2* RNA is the precursor of a new miRNA-like small RNA( mil-HongrES2) in rat epididymis

**Figure 3:**
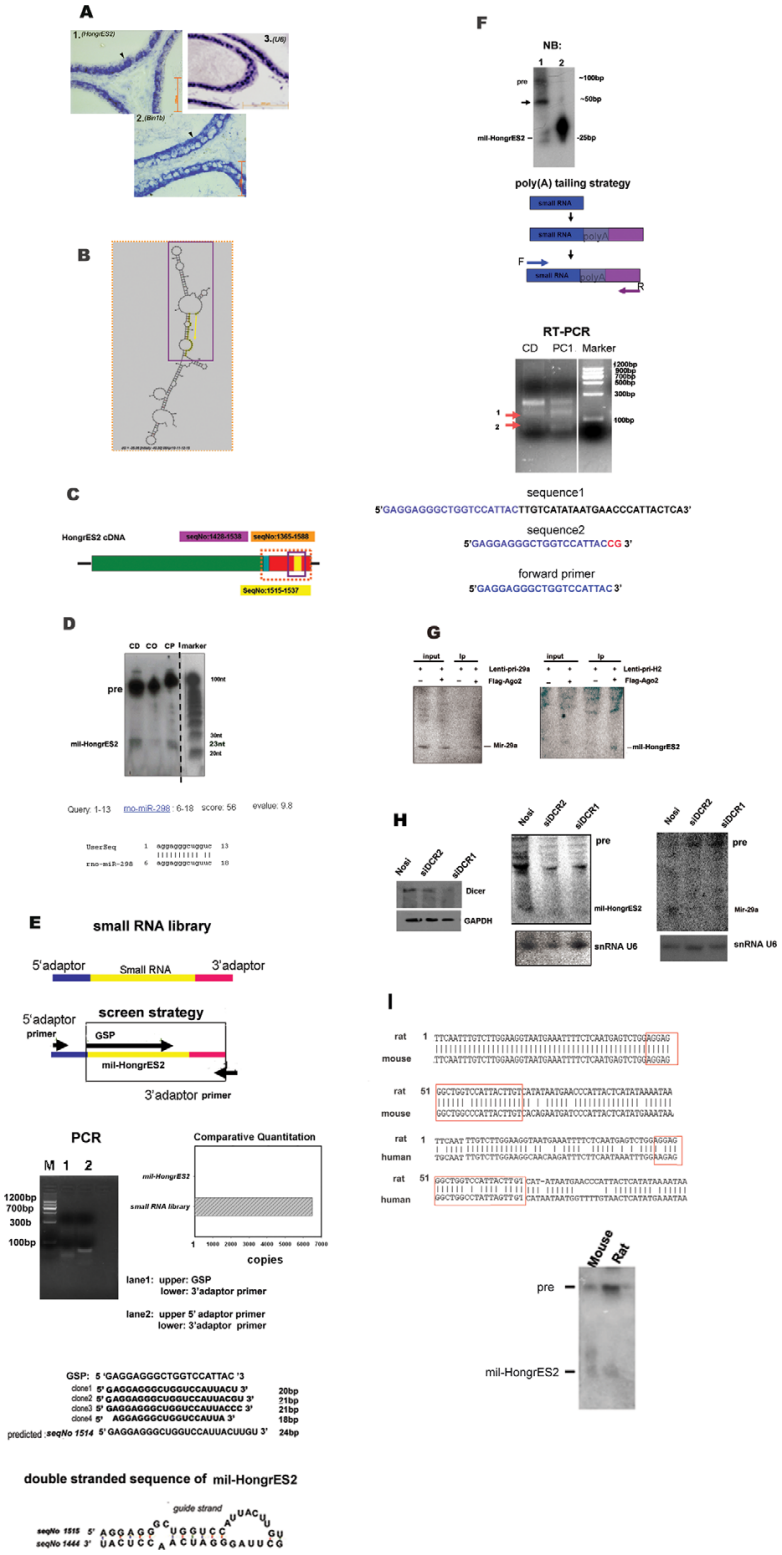
*HongrES2* RNA is the precursor of a new miRNA-like small RNA. (A) Panel 1: the *in situ* hybridization shows the subcellular localization of the 1.6-kb *HongrES2* transcript in the cauda region. Panel 2: the *in situ* hybridization shows the cytoplasmic localization of the Bin1b transcript in the caput region as a control (100×); bar = 125 µm. Panel 3: the *in situ* hybridization shows the nuclear location of U6 RNA in the corpus region as a positive control (40×); bar = 200 µm. (**B**) The secondary structure prediction of the *HongrES2* RNA by mfold. The predicted stem loop structure and mature sequence are labeled with purple and yellow, respectively. (**C**) A schematic representation of the different cDNA fragments described in (B). (**D**) Northern blot analysis showing three forms of the *HongrES2* RNA in the rat epididymis. CD: cauda; CO: corpus; CP: caput; marker: Ambion small RNA marker (10 nt–100 nt). The marker lane was exposed for a shorter time on the same membrane. The blastn search result of the *mil-HongrES2* sequence against the miRBase database. (**E**) Upper panel: depiction of how the mil-HongrES2 was bailed from the small RNA library by selective primers (GSP&3′adaptor primer). Low pannel: the *mil-HongrES2* sequences identified from the small RNA library and the miRNA:miRNA* like duplex sequence. Real-time PCR showed the camparative quantitation of the *mil-HongrES2* molecules in the whole small RNA library. (**F**) *HongrES2* is processed into *mil-HongrES2* in PC1 cells. Lane1: RNA from PC1 cells transfected with the *HongrES2* expression vector. Lane2: The 25 nt RNA oligo nucleotide used as a positive control and the size marker for the processed product. The strong band below pre form of *mil-HongrES2* pointed out by arrow was analysed through poly(A) tailing PCR using the gene specific forward primer. (**G**) Northern blotting to detect *mil-HongrES2* after anti-FLAG-Ago2 immunoprecipitation. The right panel shows PC1 cells that were transfected with HongrES2 expression plasmids alone (mock) or in combination with plasmids that express FLAG-tagged Argonaute2 (Flag-Ago2). The left panel shows a control experiment using a probe of a known mir-29a to demonstrate that the IP worked. (**H**) The left panel shows that the Dicer protein level was reduced by two different siRNAs. The right panel shows that *mil-HongrES2* expression was inhibited in DCR knockdown cells. *Mir-29a* expression was also detected as a control to show that the whole RNAi experiment was effective. Nosi: control siRNA. siDCR1/2: two different sequences of the siRNA-targeting mouse DCR gene. (**I**) Upper panel :sequence alignment of the 100-bp conserved sequence between different species of rat, mouse, and human around the *mil-HongrES2* encoding region. Red rectangles labeled out the *mil-HongrES2* sequence. Lower panel: northern blot analysis of mouse *mil-HongrES2* expression, using the rat LNA probe of *mil-HongrES2* (24 nt). Hybridization was carried out at 42°C.

*HongrES2* was investigated as RNA-coding transcript because no ideal ORFs could be found in the cDNA. *In situ* hybridization of the *HongrES2* RNA was performed. Small nuclear RNA (SnRNA) U6, a known nuclear RNA, was used as a positive control, and a known cytoplasm localization gene, Bin1b was used as another control probe[35], [36]. Comparaing the expression pattern of *HongrES2* with those of snRNA6 and Bin1b RNA, the signal localization of *HongrES2* was found to be more similar with the SnRNA6 than the Bin1b.This suggested that *HongrES2* RNA might be prone to gathering around the nuclear area. In previous reports, pri-miRNAs were considered to have the nuclear localization traits [37], [38], [39], suggesting that *HongrES2* might be a potential primary microRNA (Figure. 3A).

Further attempts to predict the secondary structure of the 3′ end, 224 bp (seqNo 1365–1588) fragment of *HongrES2* were carried out using the mfold program. The fragment seqNo 1428–1538 from chromosome 19 (with purple label in Figure 3) was predicted to fold into a stem-loop hairpin structure that lack perfect Watson-Crick complementarity. The seqNo 1515–1537 (yellow box in Figure 3) indicated the guide strand of the predicted 24-nt, mature microRNA generated by *HongrES2* (Figures. 3B and 3C). Northern blot analysis was used to investigate whether or not *HongrES2* could be processed into a mature miRNA in the epididymis. Using an LNA probe with sequence complementary to the predicted mature miRNA guide sequence, a faint but distinct band of approximately 23 nt in the cauda and caput regions of the epididymis was observed (Figure. 3D). Furthermore, an intense signal of approximately 100 bp was also detected, demonstrating the existence and accumulation of its pre-miRNA form.

To confirm the size and existence of this newly discovered small RNA, independent Northern blots with the 24-nt DNA oligo marker were performed (Figure S2). During the validation for the expression of this new miRNA-like small RNA (*mil-HongrES2*) by Northern blots, its 100- bp pre-miRNA-like band was quite intense and stable, whereas the expression of the ∼23-bp microRNA-like small RNA (*mil-HongrES2*) in normal tissues was low and sometimes difficult to be detected. Thus, a BlAST search was performed against the Sanger miRbase to determine identified miRNAs with sequence similar to that of *mil-HongrES2*; *ron-mir-298* was the only one found to have about 50% homology with *mil-HongrES2* in sequence and a very low expression ratio (about 10^−4^) compared with the *mir-29a* signal in miRNA chips (data not shown) in rat epididymis (Figure 3D). Hence, the ∼23-nt band could not possibly be generated because of cross hybridization to other small RNAs, including the *ron-mir-298*, under high stringent hybridization conditions at 65°C with LNA probes having less than 50% sequence complementary.

To study the small RNAs existing in this special male organ which might have important functions during sperm maturation, a small RNA (18–25 nt) library of rat epididymis was constructed by JS Zhang (paper in revise). However, the *mil-HongrES2* sequence was not screened by PCR amplification using 3′ and 5′ adaptor primers. Considering the low expression demonstrated by the Northern blots, it was expected that small RNAs with low copy number similar to *mil-HongrES2* would not be included in such a small-sized library, that totally no more than fifty different small RNA sequences were achieved including the known and new miRNAs, siRNAs, and some piRNA like small RNAs.

Thus, the selective primer of *mil-HongrES2* (GSP) was used as an upper primer and the 3′ adaptor primer was selected as the lower primer to bail the *mil-HongrES2* sequence in this small RNAs library (Figure 3E, upper panel).

The PCR band was cloned and sequenced. Four positive clones were acquired and compared with the sequence predicted from the stem–loop structure, The 3′ terminus of the *mil-HongrES2* around the uracil (seqNo1535 of *HongrES2*) was found to possibly have had some variations. One of the four clones(clone4) was an 18-bp core sequence of mil-HongrES2 (5′AGGAGGGCTGGTCCATTA 3′ ) inserted right between the upper and lower primers, meaning that not only a single small RNA was ligated into the 5′linker adaptor and 3′linker adaptor when the library was constructed. This situation was comparatively rare but quite possible. This sequence, screened independently of the upper primer, ascertained the 5′terminal of mil-HongrES2, which might start from the adenine (seqNo1515 of HongrES2) rather than the predicted guanine. Real-time PCR was carried out to demonstrate that the copy number of *mil-HongrES2* was so low in the constructed small RNA library that there was about only one copy molecule among six thousand copies was produced (Figure. 3E).

To confirm further that *HongrES2* could be the primary transcript of this novel ∼23-nt miRNA- like small RNA, and to ruled out the possibility that the low expression signal detected by Northern blotting was due to cross hybridization, a plasmid expressing the full-length *HongrES2* RNA (pcmv-tag4-H2) was transfected into the mouse epididymal cell line, PC1[25], which did not express any endogenous *mil-HongrES2* (Figure S2). Forty-eight hours after transfection, the extra band corresponding to the *mil-HongrES2* was detected by Northern blot (Figure. 3F, upper panel). Another strong band at ∼50-bp position was found below the ∼100-bp pre*-*form. RT-PCR was performed with the poly(A) tailing strategy to check the small RNAs generated in these transfected cells (Figure 3F middle panel). The RT-PCR bands of the ∼100-bp and ∼120-bp were cloned and sequenced. The sequencing results showed that the smaller product was the 22-bp fragment of the *mil-HongrES2* sequence (2-bp swing at the 3′terminal), and the larger product was the 45-bp fragment of the constant *HongrES2* sequence (seq. No 1514–1558) including the 23-bp *mil-HongrES2* and the 22 bp sequence behind it (Figure 3F lower panel). We guess it probably be the inter-mediated product detained during *mil-HongrES2* maturation.

To determine whether or not this small RNA was generated through a miRNA pathway-dependent process and RISC association, the PC1 cells were co-transfected with the *HongrES2* expression vector (plenti-H2-2) and a FLAG-tagged-Ago2 construct. Northern blot analysis showed that the pre- and mature forms of *HongrES2* were not only generated in the transfected cells, but were also bound to the FLAG-Ago2 protein that was immunoprecipitated by an anti-FLAG antibody conjugated with Sigma M2 beads. The results indicated that Argonaute2 not only bound this small RNA, but it was also probably involved in earlier steps of its biosynthesis, which was consistent with the recently reported dual role of the Argonaute proteins[40]. A simultaneous control experiment was carried out with the pri-mir-29a expression vector instead of the *HongrES2* expression vector to determine the specificity of the IP experiments (Figure. 3G).

The dependence of the maturation of *mil-HongrES2* on DCR was investigated by co-transfecting the PC1 cells with the *HongrES2* expression vector (plenti-H2-2) and two different siRNAs (siDCR1 and siDCR2) that target mouse Dicer1 mRNA. Western blot analysis showed that both siRNAs depleted the Dicer protein and siDCR1 was a little bit effective. The *mil-HongrES2* expression in Dicer knockdown cells was tested by Northern blotting. The results revealed that *mil-HongrES2* was slightly decreased in the Dicer-deficient cells. Meanwhile, no ∼100-bp pre-form acummulation was found in both of the Dicer knowckdown cells, which was beyond our expectation. To confirm that this phenomena was not due to the special cell line or the transfections operation, an unrelated microRNA, *mir-29a*, was tested as a control. *Mir-29a* was known to be endogenous in the PC1 cells and the Northern blot analysis showed that its expression was inhibited in both cells with siDCR RNAi as well, and its pre-miRNA forms were accumulated. These findings indicated that the biosynthesis of *mil-HongrES2* might not directly relied on the actions of Dicer as a prototypical microRNA (Figure. 3H).

Sequence analysis (BlAST search) was performed using the mouse and human genome to determine whether or not the *mil-HongrES2* coding region showed sequence conservation between these two species. Each species had one copy, on mouse chromosome 8 and human chromosome 16, with 95% and 78% identity, respectively (Figure 3I, upper panel). A gene homologous to *mil-HongrES2* was also detected in mouse (ICR) by Northern blotting (Figure 3I, lower panel).

### Inflammation accelerated the generation of *mil-HongrES2* from *HongrES2*

**Figure 4:**
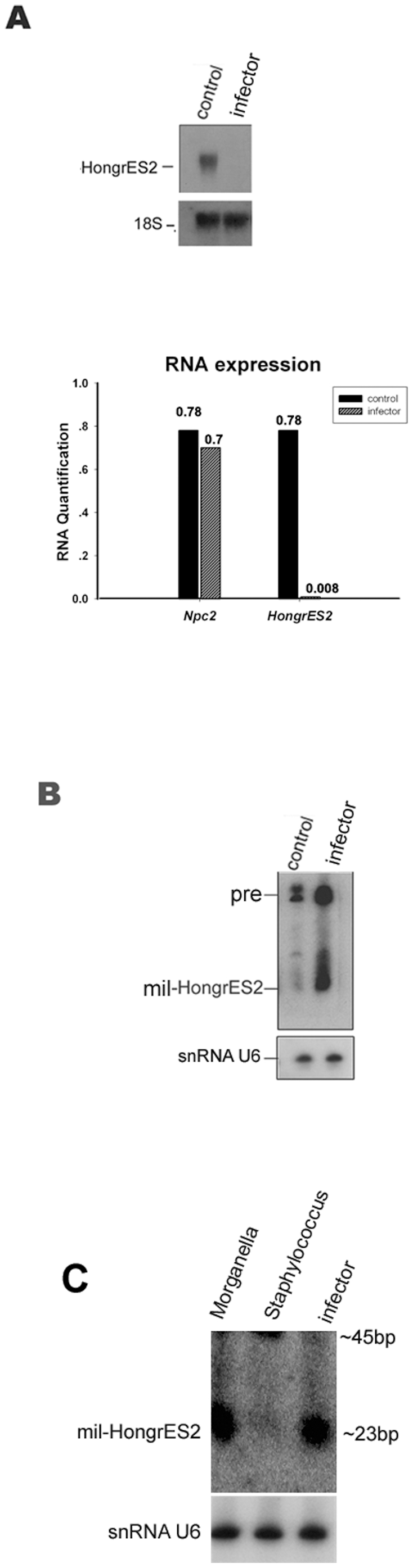
Over-expression of *mil-HongrES2* caused by inflammation of the rat epididymitis. (**A**) The upper panel showed Northern blot analysis of the RNA expression of *HongrES2*.18S rRNA was used as the loading control. The lower panel was the Real-time PCR analysis of *Ncp2* besides *HongrES2*. (**B,C**) Northern blot analysis to detect the *mil-HongrES2* expression. U6 probe was used on the stripped membrane as an internal loading control.

Interestingly, the *mil-HongrES2* abundance was very low whereas its pre form remained high.Comparing Figure 2B and Figure 3D, the processing of *mil-HongrES2* seemed to be controlled in a region-specific manner in the adult rat epididymis.This behavior was somewhat simliar with the previous report where ubiquitously expressed precursor miRNAs were processed to mature miRNA in a tissue and /or cell specific manner, in the these studies, HeLa cells expressed the miR-138–2 precursor but not the mature miR-138 because of the presence of some unknown inhibitory factor.This observation demonstrated that the regulation of pre-miRNA/mature miRNA by Dicer was extremely complicated[41], [42], [43] . Epididymitis was the most common disease in the male reproductive system that severely affected the sperm quality, the biogenesis characters of *mil-HongrES2* mentioned above suggested considering whether or not the generation of *mil-HongrES2* would be altered under a pathological context. The cauda tubules on the right-side epididymis of three adult SD rats were injected with cultured pathogenic bacteria from human patients with epididymitis. A 0.9% saline solution was used as a negative control on the left side of the organs without cut the scrotum open. Four days after injection, the animals were sacrificed. Northern blot analysis showed that the expression of *mil-HongrES2* in the epididymis was significantly enhanced, and the expression of *HongrES2* could hardly be detected in the infected group (the tissues were pooled together). In the control group, the expression of *mil-HongrES2* was much lower than in the infected group, and the *HongrES2* RNA remained detectable by Northern blot analysis (Figure. 4A and 4B). Other epididymal genes were also tested:, unlike HongrES2, some of their expression were not excessivly reduced by the bacteria infection (Figure S3).Real-time PCR analysis indicated that the amount of Ncp2 mRNA[44] was generally unchanged in the infector group (Figure. 4A, lower panel).These results suggest that the processing of *HongrES2* and the amount of *mil-HongrES2* are controlled in the physiological state and can be altered by pathological changes. Furthermore, a sample of the pathogenic bacteria from human patients, which were used to induce the over-expression of *mil-HongrES2* was sent for fractional cultivation. The results showed that the bacteria were mainly composed of two types: *Staphylococcus* and *Morganella.* These bacteria were cultured and injected into the cauda region of rat epididymis respectively. Northern blot analysis showed that just like the complex pathogenic infector from human patients mentioned above, the pure cultured *Morganella* germ could also induce the over expression of *mil-HongrES2* in rat epididymis, whereas the less harmful bacteria *Staphylococcus* could not (Figure 4C).

The epididymis protects the sperm from outside invasion of all kinds of pathogens and epididymitis is a most common disease of this organ. The over expression of the *mil-HongrES2* under epididymitis confirmed the existence of this newly defined small RNA, and provided indications of its possible anti-infection functions *in vivo* as well.

From the results above, this small RNA of *mil-HongrES2* was found to exhibited some expression and biogenesis features quite similar to a microRNA, as well as a few properties different from a typical miRNA. First, the expression of its primary form (*HongrES2)* was more stable and higher than the usual pri-miRNAs, which could hardly be detected and identified. Second, the ∼100-bp pre form size of *mil-HongrES2* was much bigger than that of typical pre-microRNAs which were usually about 60∼80 bp in animals. Third, the extra band of the 45-bp pre-form of *mil-HongrES2* repeatedly appeared in the transfected PC1 cells and it occasionally appeared in the normal rat epididymis tissues too. Fourth, it was not quite consistent with the biogenesis criteria widely accepted as a microRNA that no detection of increased precursor accumulation in cells with reduced Dicer function, although recently it had been reported that some microRNAs could be generated in the pathway independent of Dicer [45], [46], [47], [48].

According to the criteria for a new microRNA validation report [11], the 23-bp small RNA(mil-HongrES2) was considered to be a new miRNA-like small RNA, with the chimeric non-coding RNA HongrES2 RNA as its primary transcript in rat epididymis.

### The epididymis-specific gene, CES7 could be down-regulated by *HongrES2* via *mil-HongrES2*

**Figure 5:**
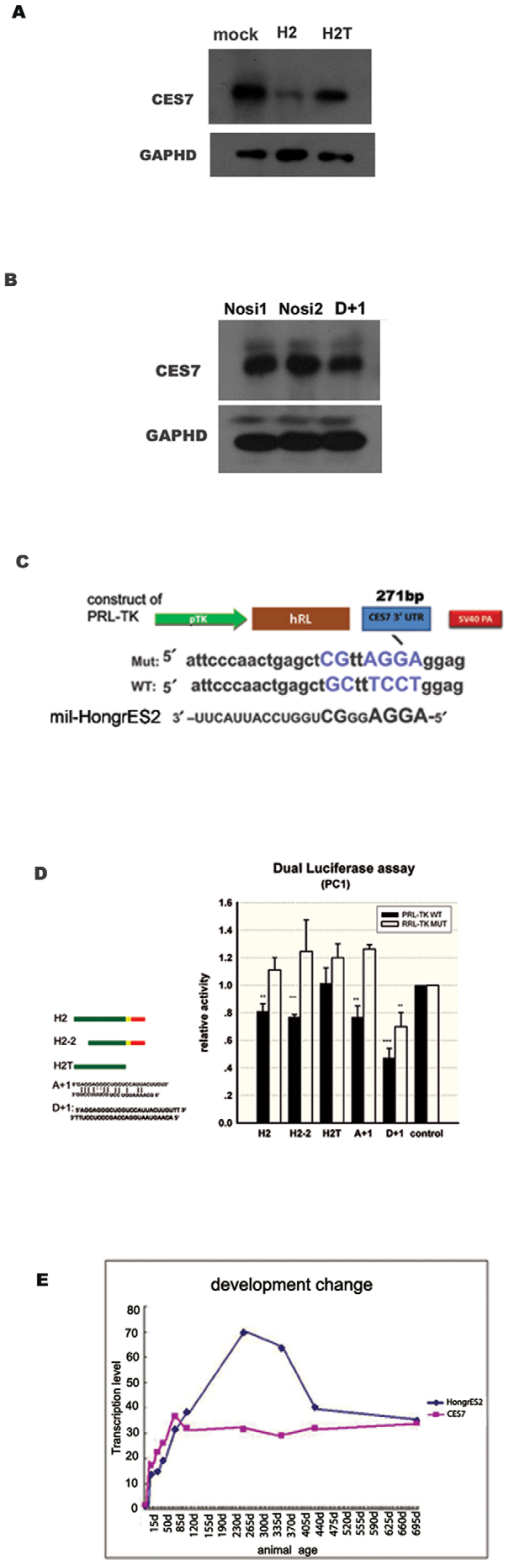
CES7 was down-regulated by *HongrES2* via *mil-HongrES2*. (**A**) Western blot indicating the amount of CES7 protein after co-transfection into PC1 cell with the CES7 expression vector and different constructs. Mock: pcmv-tag4 plasmid without insertions. H2: Pcmv-tag4a-H2. H2T: Pcmv-tag4a-H2T. (**B**) Western blot indicating the amount of CES7 protein after co-transfection into PC1 cell with the CES7 expression vector and dsRNA regulators. Nosi1 and Nosi2: negative control of unrelated dsRNAs. (**C**) Depiction of the wide-type and the target-site mutant 3′UTR of CES7 cDNA inserted into the PRL-TK luciferase reporter vector. (**D**) Dual luciferase assay activity. H2: pcmv-tag4a-H2; H2-2: plenty-H2-2; H2T: pcmv-tag4a-H2T; A+1: the imperfect duplex of *mil-HongrES2* mimics; D+1: the perfect duplex of *mil-HongrES2* mimics; the data are expressed as the mean ± SEM (N>3). **P<0.01, ***P<0.001. All of the plasmids and mimics are shown in Table 2. (**E**) Expression of CES7 mRNA and *HongrES2* RNA during the lifespan of the rat epididymis.

The sequence homology of HongrES2 and the rat CES7 gene was presented in Figure 1C. This rare phenomenon of sharing the common 3′end 216-bp sequence caused us to consider the relationship between these two genes. Non-coding RNAs (ncRNAs), both small and large, have recently gained popularity as versatile regulators of protein coding gene expression. [49], [50]. The role of *HongrES2* in regulating the expression of CES7 gene was investigated by performing co-transfection experiments in PC1 cells with the expression vector containing the full-length *CES7* (pcmv-tag4a-CES7) and the *HongrES2* expression vector (H2) as the tested group. In addition, cells co-transfected with the CES7 expression vector and pcmv-tag4a vector (mock), and cells co-transfected with the CES7 expression vector and the truncated vector comprising the *mil-HongrES2* coding region lacking the HongrES2 3′ end (H2T), were used as two different control groups. The expression of the CES7 protein was reduced by the HongrES2 expression vector (H2), as compared to the controls (mock, H2T) (Figure. 5A). These experiments were repeated independently several times, and the variation of the CES7 protein was quantified (Figure S4).

Moreover, using the synthetic mimics of *mil-HongrES2* (D+1) instead of the primary precursor(*HongrES2*), the protein level of CES7 was also found to be down-regulated compared with the negative control mimics (Figure. 5B). These experiments were repeated several times, and the variation of CES7 protein was quantified (Figure S5). All of these observations indicated that *HongrES2* could down-regulate the expression of CES7 *in vitro*, probably through the *mil-HongrES2* function.

To determine whether HongrES2 directly down-regulats the CES7 gene expression via *mil-HongrES2* by sequence base pairing, PRL-TK Renilla luciferase reporters containing either the CES7 wild-type 3′UTR or the mil-HongrES2 base pairing site mutant 3′UTR were constructed (Figure. 5C). A dual luciferase assay showed that the relative luciferase activity dropped by 20%–50% when the wild-type reporter construct was co-transfected into PC1 cells with all of the regulators necessary to generate the *mil-HongrES2*, such as the full-length HongrES2 expression vector (H2), a 5′ end, 591-bp truncated HongrES2 vector (H2-2), the perfect *mil-HongrES2* mimics duplex (D+1), and the imperfect duplex (A+1) of the *mil-HongrES2* mimics. However, the 3′ end, 216-bp truncated HongrES2 vector (H2T), which cannot generate *mil-HongrES2*, did not inhibit the activity. A reporter with mutations in the predicted *mil-HongrES2* target site was not suppressed by the regulators, except for the perfect duplex mimics (D+1), which retained an approximate 20% suppression effects (Figure. 5D). Considering the sequence homology between *CES7* and *HongrES2* mentioned above (Figure. 1C), its passenger strand would pair perfectly with the *mil-HongrES2* coding region on the 3′UTR, potentially explaining the knockdown effect.

The developmental changes in the rat *CES7* mRNA and the *HongrES2* RNA expression levels during the rat lifespan were compared by Northern blot analysis. Interestingly, the trend in their expression patterns was quite different. *CES7* mRNA expression quickly increased at an early stage (before 90 days old), and it plateaued to a stable level until two years of age, whereas the *HongrES2* RNA expression level increased for a longer time after initiation (until 270 days of age; Figure. 5F). The wave trend of the *HongrES2* RNA and *CES7* mRNA expression curve was temporally reciprocal. According to a previous report, this phenomena was coincident with the regulator-target pair relationship between *HongrES2* and *CES7* mentioned above[51]. Thus, this hypothesis was formed:, When the exprssion level of CES7 protein needs to be cut down *in vivo*, not only *CES7* mRNA expression is reduced by the organism, but the expression of *HongrES2* is increased to prohibit the CES7 protein translation process at the post-transcription level as well.

### *Mil-HongrES2* over-expression in the cauda epididymis reduced the CES7 protein level and sperm capacitation

**Figure 6:**
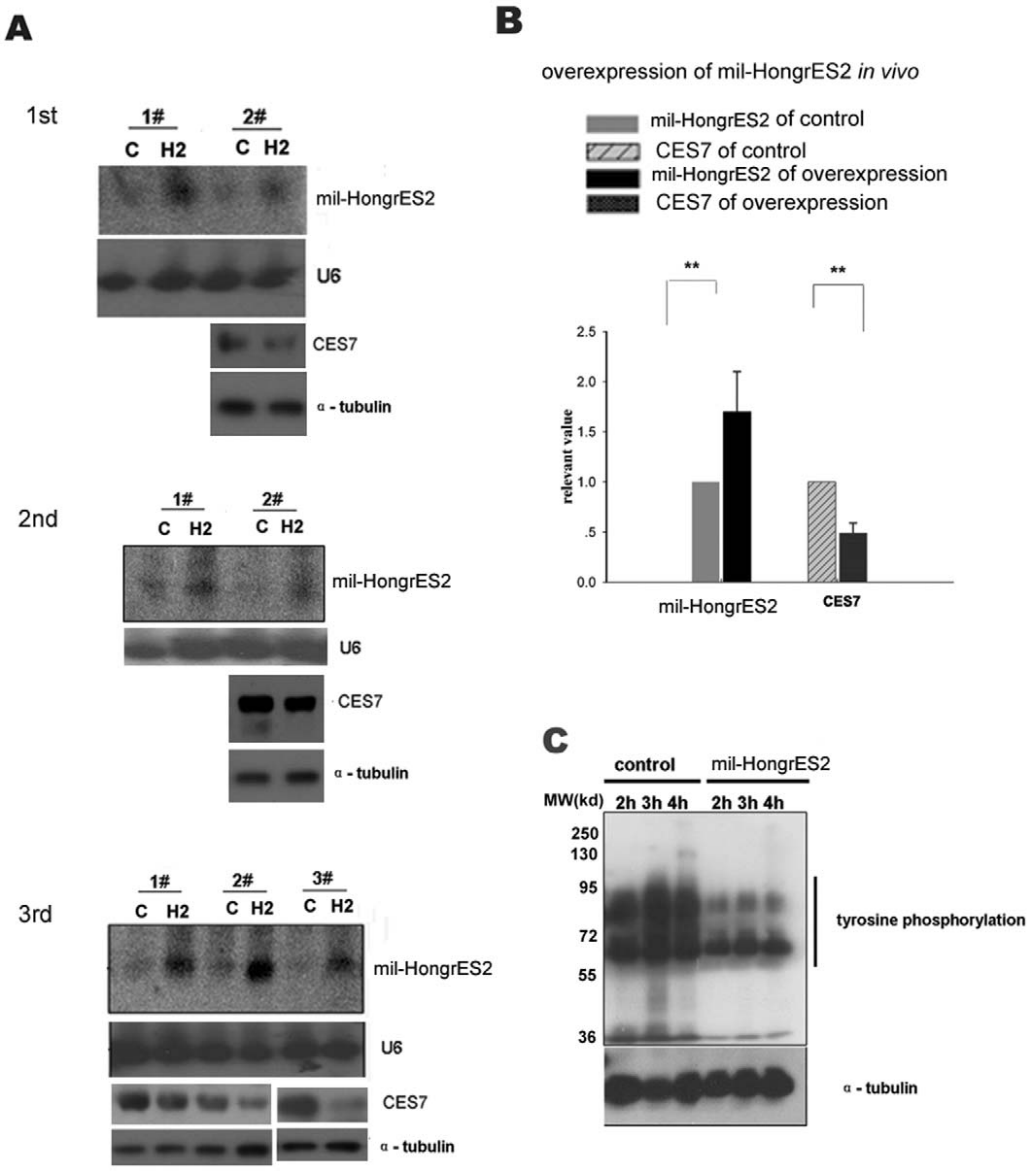
Over-expression of *mil-HongrES2* by injecting agomir into the epididymis to reduce the cauda sperm capacitation. (**A**) *Mil-HongrES2* over-expression by *mil-HongrES2* mimics agomir down-regulation of CES7 protein expression. The two upper panels show the northern blot analysis of *mil-HongrES2* over-expression. C: control group. H2: over expression group. U6 was used as the internal loading control. The number shows two or three different individuals. The two lower panels show the corresponding CES7 protein level *in vivo*, and α-tubulin was used as the loading control. The experiments were carried out independently and the results of 3 replicates are shown and indicated by the replicate number (1^st^, 2^nd^ or 3^rd^). (**B**) Quantification analysis of *mil-HongrES2* over-expression and the down-regulation of CES7 protein from (A) (n = 7 ). The data are expressed as the mean ± SEM. **P<0.001. (**C**) The change in protein tyrosine phosphorylation in *mil-HongrES2* over-expressing sperm after incubation in the sperm culture medium. Total protein from the spermatozoa was collected for western blot analysis after 2 h, 3 h and 4 h of incubation; α-tubulin was used as the loading control.

To investigate whether the amount of *mil-HongrES2* was critical to the epididymis micro-environment during sperm epididymal maturation, *mil-HongrES2* RNA was over expressed *in vivo* by directly injecting the modified *mil-HongrES2* agomir into the right side of the cauda epididymis; a “seed sequence” mutant scramble agomir was used as a negative control in the left side. Four days later, the amount of CES7 protein was decreased, whereas the amount of *mil-HongrES2* expression was elevated in the over-expression group (Figures. 6A and 6B). The *CES7* gene is a newly defined carboxylesterase with cholesterol esterase activity [32], [33]; cholesterol is very important during the sperm capacitation process [52]. Tyrosine phosphorylation of sperm proteins has been reported to be an indicator of capacitation signaling cascade activation.[53], [54], [55] [56]. Tyrosine phosphorylation of the sperm proteins was then assayed to investigate whether or not sperm capacitation would be affected when *mil-HongrES2* was overexpressed. Western blot analysis showed that tyrosine phosphorylation of the sperm protein during capacitation was affected (Figure. 6C). This result meant that the endogenously low expression of *mil-HongrES2* was important for sperm maturation in the normal rat epididymis, and that *HongrES2* RNA may play a regulatory role in sperm maturation via *mil-HongrES2* maturation.

## Discussion

In the present study, a new 1.6 kb mRNA-like chimeric non-coding RNA named *HongrES2* in adult rat epididymis was found occasionally. Its 3′terminal sequence was homologous with another epididymis specific gene, CES7. The gene was further identified and characterized to be the primary precursor of a ∼23-bp miRNA-like small RNA (*mil-HongrES2*), which could regulate the expression of CES7 gene.

A relatively long, polyadenylated transcript encoded by the *Caenorhabditis elegans* let-7 gene undergoes trans-splicing to the spliced leader 1 (SL1) RNA to generate a mature microRNA [57]. A newly defined conserved ncRNA gene Dmr (Dmrt1-related gene) in rodents was demonstrated to trans-splice with DMRT1 (Doublesex-related transcription factor) recently. Dmr was demonstrated to serve mainly as a 3′ UTR, which promotes trans-splicing to produce a novel chimeric transcript (Dmr-Dmrt1), down-regulating the Dmrt1 protein expression [58]. DMRT1, which was reported to function in coordinating spermatogonial development and mitotic amplification with meiosis, is considered as an important gene involved in spermatogenesis. Therefore, the chimeric transcript (Dmr-Dmrt1) was presumed to play a negative regulatory role in male sexual regulation [59]. These facts suggested the possibility that both the conding gene and the noncoding gene could serve as a 3′UTR to trans-splice to produce a novel chimeric transcript (coding or noncoding) to increase the diversity of gene functions; the mechanism for this, however, is not yet clear. Wang's group found that approximately half of the chimeric RNAs have short homologous sequences (SHSs) at the junction sites that are essential for generating a novel kind of chimeric RNA. In the present study, an SHS (CTGCTTT) was found between the two fragments from different chromosomes. However, this sequence did not match any of the previously reported consensus sequences [21]. This peculiar chimeric, non-coding RNA formed by the 216-bp 3′fragment of the *HongES2* originating from the coding sequence, and the 1372-bp 5′-fragment of the *HongES2* originating from non-coding sequence in chromosome 5 combined with an SHS bridge, provides an ideal model for gaining further understanding of the transcriptional regulation of precursors of small RNAs.

*Mil-HongrES2* was firstly hypothesized to be a microRNA because of its length and the pre-miRNA like fold-back structure (Figure 3B). However, the stem-loop structure did not have the typical characteristic feature of a pre-miRNAs, as reported previously, for which the guide strand should be located on the stem region of about 30–36 bp [60]. Among the three main categories of small RNAs (microRNAs, siRNAs and piRNAs) in animals, *mil-HongrES2* was most similar to the features of the microRNAs, as siRNAs can be processed from a broader range of duplex structures that are perfectly base paired, or nearly so, and the structure of the piRNAs precursor were not well defined but are apparently single-stranded.

Following the discovery of miRNAs, researchers have started searching for their targets and uncovering the biological functions of individual miRNAs[61], [62]. However, less attention has been paid to the sub-classification of these miRNAs. The most important criteria for annotating a cloned sequence as a miRNA are their characteristic length (_22 nucleotides) and a compact pre-miRNA fold-back structure [11]. Furthermore, miRNAs generally adhere to additional properties, including precise 5′end processing, asymmetric strand accumulation, and sequence conservation. Surprisingly, not all miRNA sequences comply with these criteria. A recent study reported the miRNAs to be sub-divided into four categories, namely, “prototypical,” “repeat-clustered,” “repeat-derived,” and “unclassified” miRNAs [63]. MiRNAs were considered prototypical if they met defined criteria regarding their 5′ end processing, lack of repetitiveness, and cross-species conservation. Overall, 59% of all miRNA genes were classified as prototypical. Repeat-clustered and repeat-derived miRNAs originate from highly repetitive genomic sequences, either clustered or dispersed. In contrast to prototypical miRNAs, precursors of repeat-clustered miRNAs did not preserve the strand asymmetry between sequence-related precursors. The remaining 28% of the miRNA genes were termed unclassified, their products showed irregularities in processing and/or unusual sequence variations including deletions and variation in the seed sequence between human and rodent orthologs [64]. Only 17% of all miRNA sequences identified by a recent deep-sequencing study were prototypical [65]. The remaining miRNAs, though clearly originating from hairpin precursors, showed unusual maturation or sequence conservation patterns. For many more recently reported miRNA candidates, cloning evidence was not found. These small RNAs are speculated to originate from dsRNA structures that only accidentally enter the RNAi pathway, such as fold-back elements controlled by dsRNA deaminases or the binding sites of RNAi unrelated dsRNA-binding proteins. Thus, identification of *mil-HongrES2* might also indicate those potential unclassified miRNA candidates.

Transfection assays revealed that when the *CES7* RNA, which had the same 216-bp 3′UTR but not the 5′ 1372-bp fragment of *HongrES2*, was transfected into the PC1 cell line, no *mil-HongrES2* signal was detected by Northern blot analysis. Dual luciferase assay experiments with different types of constructs also implied that the 5′RNA fragment derived from chromosome 5, might be important for generating *mil-HongrES2* (Figure S6A). *In situ* hybridization showed that the *CES7* mRNA was located in the cell cytoplasm, which is different from the cellular localization trait of *HongrES2* (Figure S6B). The secondary structure of the 223-bp of the CES7 3′end (with 216 bp identical to *HongrES2*) was also carried out by mfold; and none of the predicted structures showed that the *mil-HongrES2* sequence could be the arm of a possible stem-loop (Figure S7). Sequences and structures flanking the miRNA hairpin affect its maturation [66]. The same mechanism might explain the observations above.

In summary, beginning from a new gene EST(*HongrES2 EST*) the full length of a new gene (*HongrES2*) was obtained. Subsequent analyses of its biological properties revealed it to be the precursor of a miRNA-like small RNA (*mil-HongrES2*), which could down-regulated the expression of CES7 both *in vitro* and *in vivo*. However, these findings are still preliminary. The over-expression of *mil-HongrES2* in the rat epididymis implied that it functions in sperm maturation. Therefore, further in vivo studies are necessary to explore the biological significance of this ncRNA in the context of sperm maturation.

## Supporting Information

Figure S1 **The sequence of the mouse 163bp probe for screen the rat epididymis cDNA library.**
(TIF)

Figure S2 **Northern blot analysis detected the mil-HongrES2 expression in rat epdidydimis.** PC1 cells and rat liver RNA were used as negative control. The 24 bp DNA oligo had complimentary sequence to the LNA *mil-HongrES2* probe was used as positive control and a precise size marker. CD total: ttl RNA of rat cauda; Cd small:small RNA of rat cauda; cell: ttl RNA of PC1 cells; Liver: ttl RNA of rat liver. marker: Ambion small RNA marker(10 nt-100 nt).
(TIF)

Figure S3 **RT-PCR analysis of different genes expressed in rat epididymis.** The results were showed in pairs, left band was the gene expression in the infection group, while the right band was in the control group.
(TIF)

Figure S4 **CES7 protein level was reduced by *HongrES2* in PC1 cells. (A)** Western blot of CES7 proteins after co-transfection into PC1 cell with CES7 and different constructs.Mock: pcmv-tag4 plasmid without insertions. H2: Pcmv-tag4a-H2. H2T: Pcmv-tag4a-H2T. 3^rd^ panel was the raw data of Figure. 5C. **(B)** Quantification analysis of the CES7 protein expression of the western blot in A. Data were expressed as the means±SEM.
(TIF)

Figure S5 **CES7 protein level was reduced by mil-HongrES2 mimics.** **(A)** Western blot of CES7 proteins after co-transfection into PC1 cells with CES7 expression vector and different dsRNA regulators. Nosi1 and Nosi2: negative control of irrelevant dsRNAs. A+1: the imperfect duplex of mil-HongrES2 mimics; D+1: the perfect duplex of mil-HongrES2 mimics. 3^rd^ panel was the raw data of figure5D. **(B)** Quantification of the CES7 protein expression of the western blot in A(1^st^,2^nd^ ,3^rd^ ). Data were expressed as the means±SEM
(TIF)

Figure S6 **CES7 mRNA could not play the role of the precursor of *mil-HongrES2*.** **(A)** Dual luciferase assay activity. CES7: pcmv-tag4a-CES7; control: pcmv-tag4a mock plasmid. **(B)** In situ hybridization of CES7 mRNA. The signal was brown and was stained in the cytoplasm of epididymal epithelium.
(TIF)

Figure S7 **The secondary structure of *CES7* mRNA 3′end 223 bp.** Yellow color labeled out the *mil-HongrES2* encoding region.
(TIF)

Table S1 **Sequences of probes, primers and siRNAs sequences.** All the probes, primers and siRNAs sequences used in the text were listed in the table according to the figures in which they were mentioned.
(DOC)

Table S2 **Constructs used in the paper.** All the constructs mentioned in the article were listed in the table.
(DOC)
