# Supplementary figures and images for: Identification and Characterization of a Novel Non-Coding RNA Involved in Sperm Maturation
Source: PLoS One. 2011 Oct 12;6(10):e26053. doi: 10.1371/journal.pone.0026053 (PMC3192136; doi:10.1371/journal.pone.0026053)

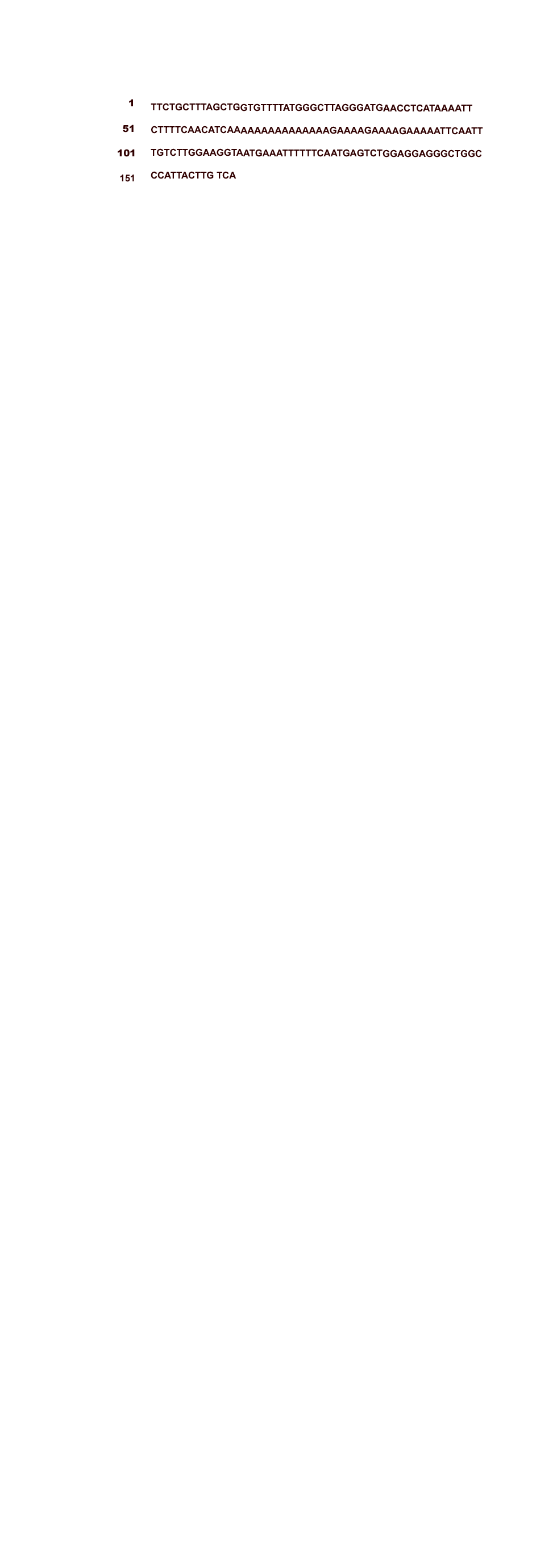

Supplement: Figure S1 — The sequence of the mouse 163bp probe for screen the rat epididymis cDNA library. (TIF) [file pone.0026053.s001.tif]

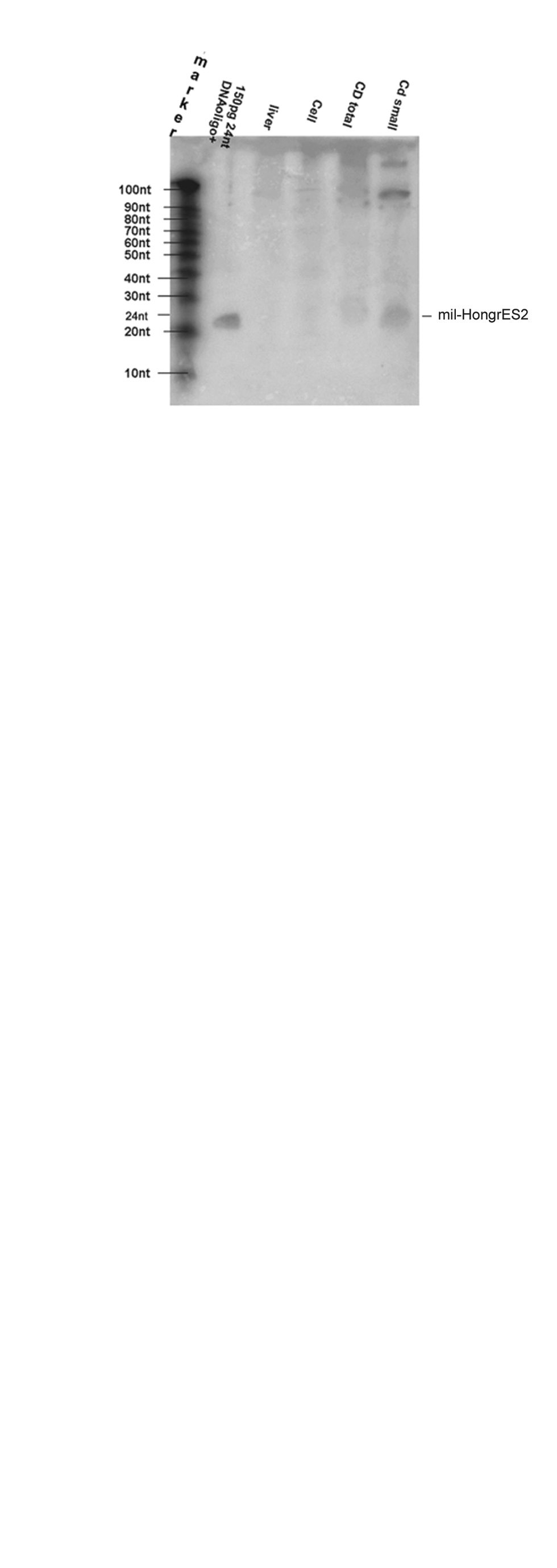

Supplement: Figure S2 — Northern blot analysis detected the mil-HongrES2 expression in rat epdidydimis. PC1 cells and rat liver RNA were used as negative control. The 24 bp DNA oligo had complimentary sequence to the LNA mil-HongrES2 probe was used as positive control and a precise size marker. CD total: ttl RNA of rat cauda; Cd small:small RNA of rat cauda; cell: ttl RNA of PC1 cells; Liver: ttl RNA of rat liver. marker: Ambion small RNA marker(10 nt-100 nt). (TIF) [file pone.0026053.s002.tif]

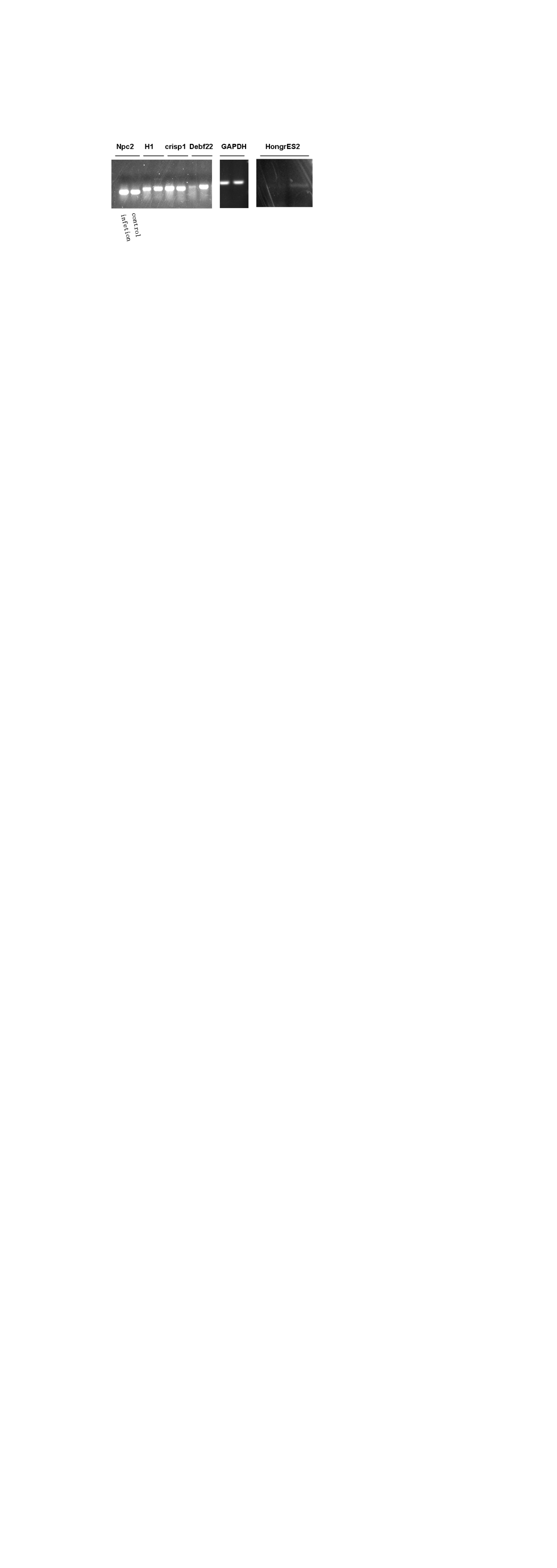

Supplement: Figure S3 — RT-PCR analysis of different genes expressed in rat epididymis. The results were showed in pairs, left band was the gene expression in the infection group, while the right band was in the control group. (TIF) [file pone.0026053.s003.tif]

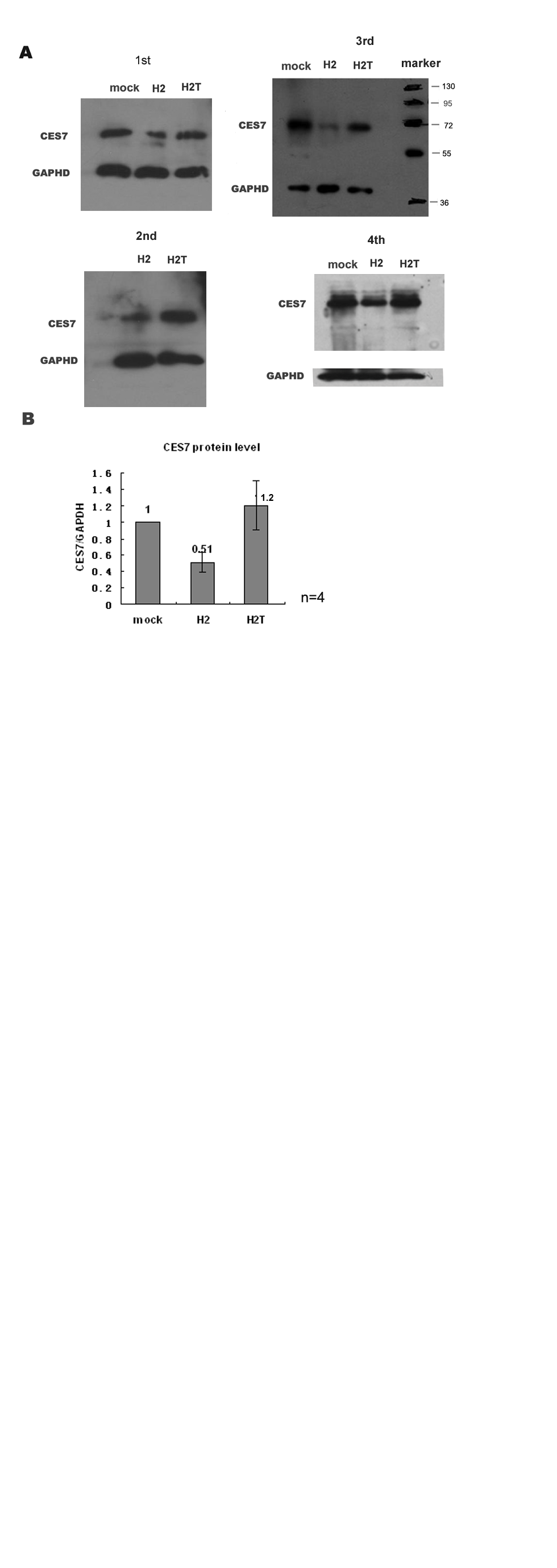

Supplement: Figure S4 — CES7 protein level was reduced by HongrES2 in PC1 cells. (A) Western blot of CES7 proteins after co-transfection into PC1 cell with CES7 and different constructs.Mock: pcmv-tag4 plasmid without insertions. H2: Pcmv-tag4a-H2. H2T: Pcmv-tag4a-H2T. 3rd panel was the raw data of Figure. 5C. (B) Quantification analysis of the CES7 protein expression of the western blot in A. Data were expressed as the means±SEM. (TIF) [file pone.0026053.s004.tif]

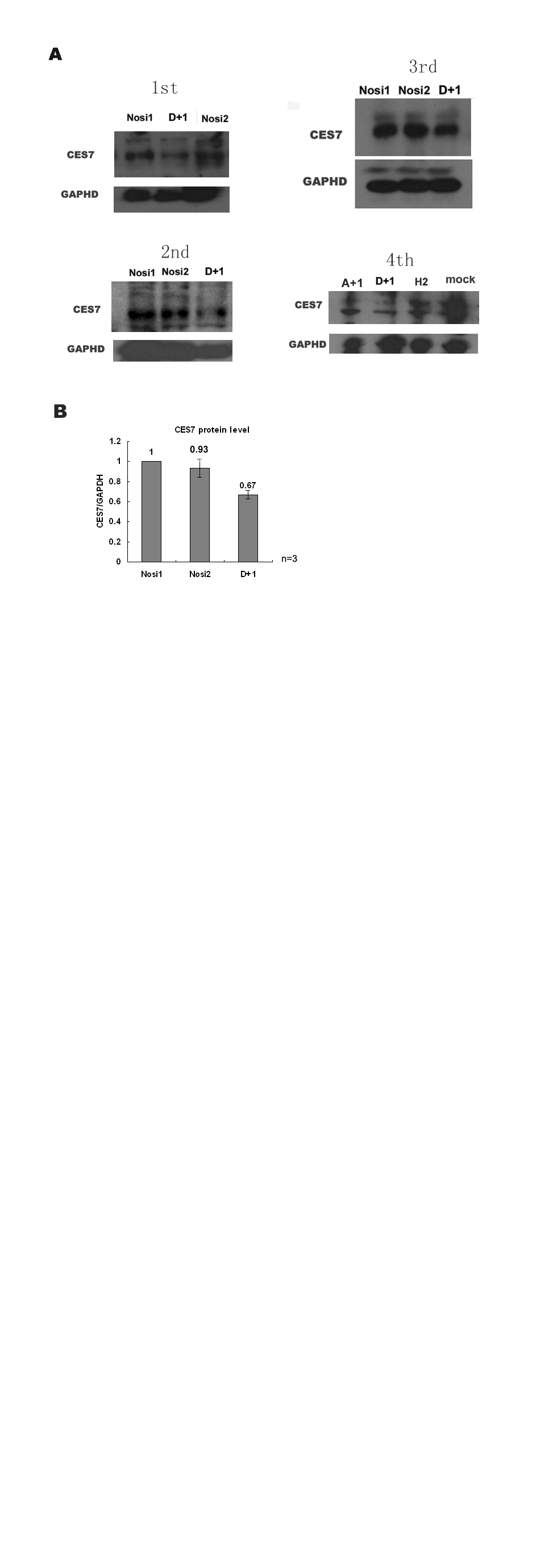

Supplement: Figure S5 — CES7 protein level was reduced by mil-HongrES2 mimics. (A) Western blot of CES7 proteins after co-transfection into PC1 cells with CES7 expression vector and different dsRNA regulators. Nosi1 and Nosi2: negative control of irrelevant dsRNAs. A+1: the imperfect duplex of mil-HongrES2 mimics; D+1: the perfect duplex of mil-HongrES2 mimics. 3rd panel was the raw data of figure5D. (B) Quantification of the CES7 protein expression of the western blot in A(1st,2nd ,3rd ). Data were expressed as the means±SEM (TIF) [file pone.0026053.s005.tif]

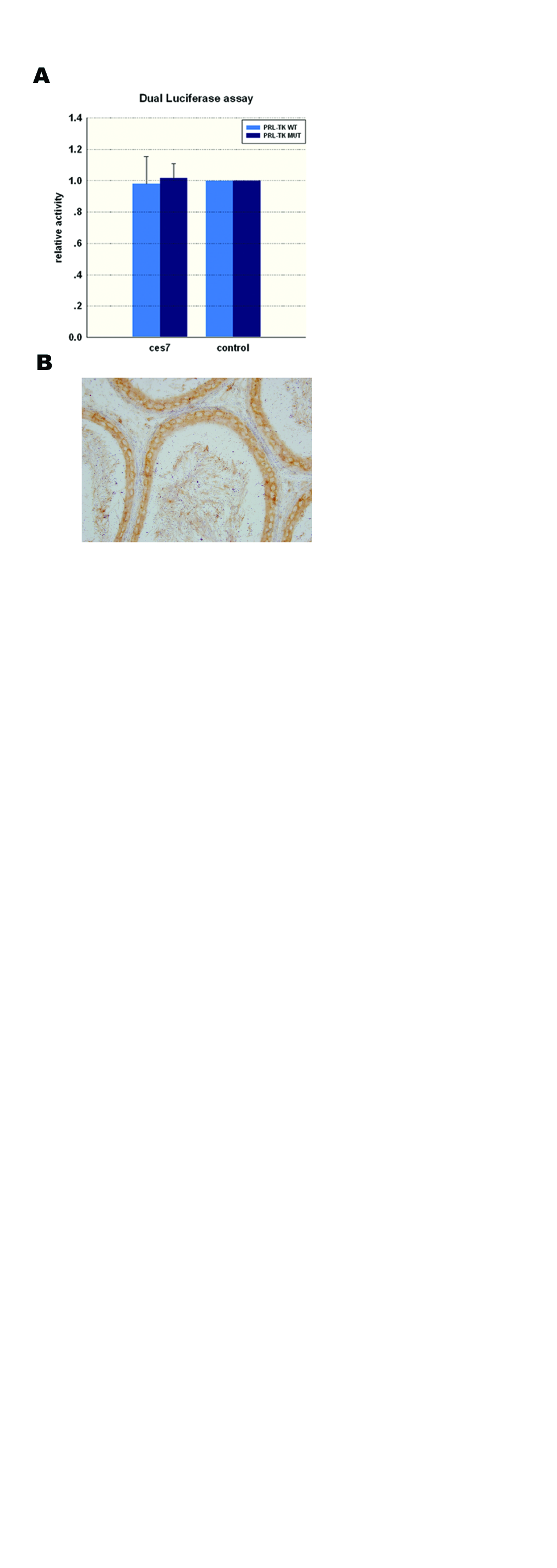

Supplement: Figure S6 — CES7 mRNA could not play the role of the precursor of mil-HongrES2. (A) Dual luciferase assay activity. CES7: pcmv-tag4a-CES7; control: pcmv-tag4a mock plasmid. (B) In situ hybridization of CES7 mRNA. The signal was brown and was stained in the cytoplasm of epididymal epithelium. (TIF) [file pone.0026053.s006.tif]

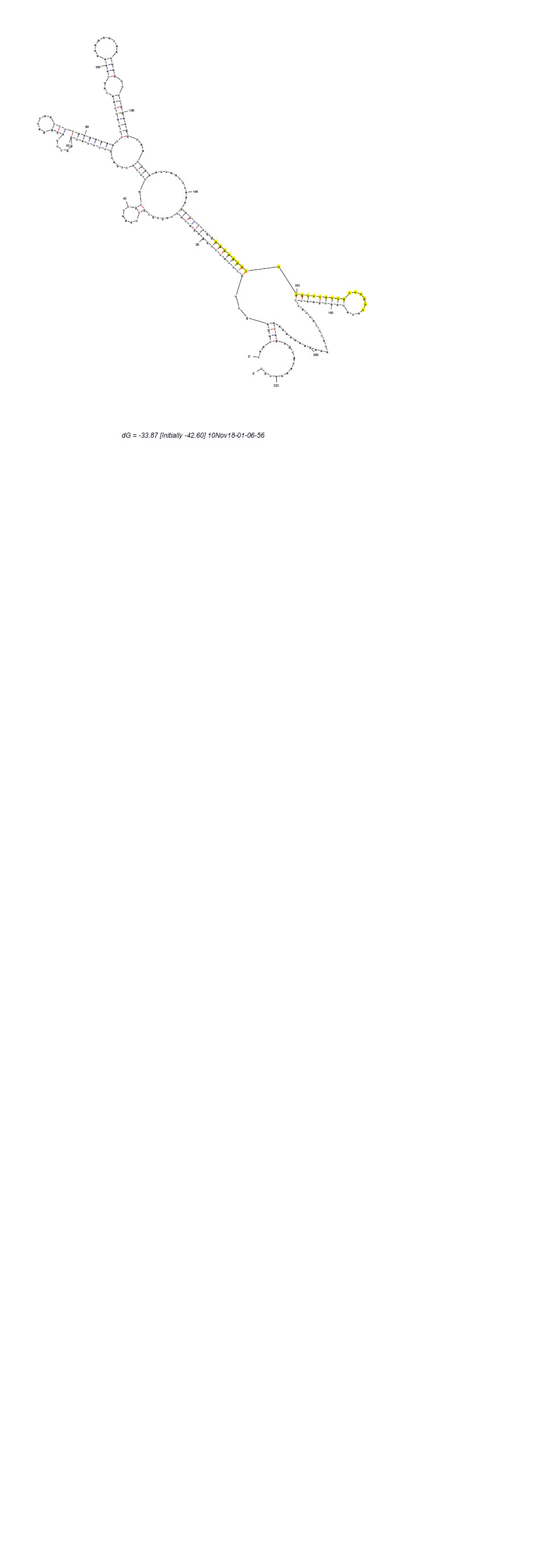

Supplement: Figure S7 — The secondary structure of CES7 mRNA 3′end 223 bp. Yellow color labeled out the mil-HongrES2 encoding region. (TIF) [file pone.0026053.s007.tif]
