# Supplementary material for: Identification and Characterization of a Novel Non-Coding RNA Involved in Sperm Maturation
Source: PLoS One. 2011 Oct 12;6(10):e26053. doi: 10.1371/journal.pone.0026053 (PMC3192136; doi:10.1371/journal.pone.0026053)
Supplement: Table S1 — Sequences of probes, primers and siRNAs sequences. All the probes, primers and siRNAs sequences used in the text were listed in the table according to the figures in which they were mentioned. (DOC) [file pone.0026053.s008.doc]

**Table1 Sequences of probes, primers and siRNAs sequences**

| Probes | Sequence / length | seqNo on the gene |
| --- | --- | --- |
| **Fig1B,C;Fig2(A-E);Fig3A;Fig4A** |  |  |
| probe1 | 504bp | seqNo861-1364(HongrES2,) |
| **Fig1B, C , Figure S6** |  |  |
| probe3 for in-situ and Northern blot | 1191bp | seqNo498-1689 (CES7) |
| **Fig1B,C** |  |  |
| probe2 | 1978bp | seqNo116-2094(CES7) |
| **Fig3A** |  |  |
| Bin1b in-situ probe | 280bp | seqNo1-280(Bin1b) |
| **Fig3D, F; Fig4B,C,** |  |  |
| LNA probe of mil-HongrES2(24nt) | 5’acaagtaatggaccagccctcctc 3’ | seqNo1514-1537(HongrES2) |
| **Fig3G,H** |  |  |
| LNA probe of mil-HongrES2(21nt) | 5’aagtaatggaccagccctcct 3’ | seqNo1515-1535(HongrES2) |
| **primers** |  |  |
| **Fig1A** |  |  |
| GSP2 for 5’RACE (1^st^ around) | 5’ggatcaattcccagcatggcagtt3’ | seqNo250-274(HongrES2) |
| GSP1 for 5’RACE(1^st^ around) | 5’ggatcccacaccttcacacagacat3’ | seqNo303-326(HongrES2) |
| GSP1 for 5’RACE (2^nd^ around) | 5’tcgccttgattactctgtctttga 3’ | seqNo149-172(HongrES2) |
| GSP2 for 5’RACE (2^nd^ around) | 5’gtctttgatctaagaactgaccagg 3’ | seqNo132-156(HongrES2) |
| GSP3 for 5’RACE (2^nd^ around) | 5’cccagagtgatataaaagcagactg3’ | seqNo94-118(HongrES2) |
| GSP1 for 3’RACE | 5’ctgacagcatcctttccatc 3’ | seqNo861-879(HongrES2) |
| GSP2 for 3’RACE | 5’aattgcttcctgttttcccctgtat 3’ | seqNo1234-1258(HongrES2) |
| **Fig1B,Fig2A,B,C,D,E** |  |  |
| Upperfor T-easy-H2  (for probe1): | 5’ctgacagcatcctttccatc 3’ | seqNo861-879(HongrES2) |
| Lower for T-easy-H2 | 5’ttaacggactgtaagtggaa 3’ | seqNo1364-1343(HongrES2) |
| **Fig2B** |  |  |
| Upper primer for QPCR: | 5’agggaaactttttcaaaagc 3’ | seqNo330-349 (HongrES2) |
| Lower primer for QPCR: | 5’ttgaagaaccactagaacca 3’ | seqNo487-506(HongrES2) |
| **Fig1D** |  |  |
| Upper primer lane1 | 5’agggaaactttttcaaaagc 3’ | seqNo330-349 (HongrES2) |
| Upper primer lane2 | 5’ctgacagcatcctttccatc 3’ | seqNo861-879(HongrES2) |
| Common lower primer | 5’acattttgcactctgattttatttt 3’ | seqNo1563-1587(HongrES2) |
| nested pcr primer 1^st^ around for lane1 | 5’gaagtagtaaaagatcacacaaggt3’ | seqNo171-195(HongrES2) |
| **Fig3E** |  |  |
| 5’adaptor primer | 5’ atcgtaggcacctgaaa 3' |  |
| 3’adaptor primer | 5’ attgatggtgcctacag 3’ |  |
| **Fig3F,G,H** |  |  |
| upper for pcmv-tag4a-H2 | 5’ccgaattccaaaactataccatgacttca | seqNo38-62(HongrES2) |
|  | gccg 3’ |  |
| lower for pcmv-tag4a-H2 | 5’cgctctcgagacattttgcactctgatttta | seqNo1563-1587(HongrES2) |
|  | tttt 3’ |  |
| **Fig5C,D,E** |  |  |
| upper for pcmv-tag4-H2T | 5’ccgaattccaaaactataccatgacttca | seqNo38-62(HongrES2) |
|  | gccg 3’ |  |
| lower for pcmv-tag4-H2T | 5’cgctctcgagaaggtgaatgtcaggcaa | seqNo1346-1364(HongrES2) |
|  | tt 3’ |  |
| upper for pcmv-tag4-CES7 | 5’cggaattcatgagtggggactgggtg3’ | seqNo116-133(rat CES7) |
| lower for pcmv-tag4-CES7 | 5’cgcaagctttgggttcattatatgacaa3’ | seqNo2075-2094(rat CES7) |
| upper for PRL-TK-wt | 5’ tctagatgcttctgattttcttctaag 3’ | seqNo1858-1878(rat CES7) |
| lower for PRL-TK-wt | 5’gcggccgccattttgcactctgattttattt | seqNo2105-2126(rat CES7) |
|  | t 3’ |  |
| upper PRL-TK-mut | 5’cccaactgagct***cgttagga***ggagactt | seqNo1905-1936(rat CES7) |
|  | ctgt 3’ |  |
| lower PRL-TK-mut | 5’acagaagtctcctcctaacgagctcagtt | seqNo1905-1936(rat CES7) |
|  | ggg3’ |  |
| **siRNAs,mimics,agomirs** |  |  |
| **Fig3H** |  |  |
| siDCR1 | 5’acaucaaggugcuacuagatt 3’ | seqNo3651-3669 |
|  | 5’ucuaguagcaccuugaugutt 3 | (Dicer1 mus) |
| siDCR2 | 5’aaggcuuaucuucugcaggcutt 3’ | seqNo5269-5289 |
|  | 5’agccugcagaagauaagccuutt 3’ | (Dicer1 mus) |
| Nosi | 5’uucuccgaacgugucacgutt 3’ | 16 base overlap with |
|  | 5’acgugacacguucggagaatt 3’ | Thermotoga , No other match |
| **Fig5E,D** |  |  |
| D+1 (2’-O-methyl-RNA synthesis) | 5’aggagggcugguccauuacuugutt | sense seqNo(1515-1537) |
|  | 5’acaaguaauggaccagcccuccutt | antisense: complete pair |
| A+1  (2’-O-methyl-RNA synthesis) | 5’gaggagggcugguccauuacuugu  5’gcaaaauguccugcuuuccug 3’ | sense seqNo(1514-1537)  antisense:incomplete pair |
| **Fig6C** |  |  |
| Mil-HongrES2 agomir | 5'aggagggcugguccauuacuu 3' | with modification |
| Control agomir | 5'acctcccgugguccauuacuu 3' | with modification |
| upper for pcmv-tag4-CES7 | 5’cggaattcatgagtggggactgggtg3’ | seqNo116-133(rat CES7) |
| lower for pcmv-tag4-CES7 | 5’cgcaagctttgggttcattatatgacaag | seqNo2075-2094(rat CES7) |
| upper for PRL-TK-wt | 5’ tctagatgcttctgattttcttctaag 3’ | seqNo1858-1878(rat CES7) |
| lower for PRL-TK-wt | 5’gcggccgccattttgcactctgattttattt | seqNo2105-2126(rat CES7) |
| upper PRL-TK-mut | 5’cccaactgagct***cgttagga***ggagactt | seqNo1905-1936(rat CES7) |
|  | ctgt 3’ |  |
| lower PRL-TK-mut | 5’acagaagtctcctcctaacgagctcagtt | seqNo1905-1936(rat CES7) |
|  | ggg 3’ |  |
