# Supplementary material for: Identification and Characterization of a Novel Non-Coding RNA Involved in Sperm Maturation
Source: PLoS One. 2011 Oct 12;6(10):e26053. doi: 10.1371/journal.pone.0026053 (PMC3192136; doi:10.1371/journal.pone.0026053)
Supplement: Table S2 — Constructs used in the paper. All the constructs mentioned in the article were listed in the table. (DOC) [file pone.0026053.s009.doc]

Table2 constructs

| Vector name | Insertion fragments | Monoclonal site used |
| --- | --- | --- |
| T-easy-H2 | 504bp,seqNo861-1364,HongrES2 | none |
| T-easy-CES7 | 1191bp seqNo498-1689,CES7 | none |
| T-easy-bin1b | 280bp,seqNo1-280 rat bin1b | none |
| Pcmv-tag4a-H2 | 1550bp,seqNo38-1587 HongrES2 | EcoRI ,Xhol |
| Pcmv-tag4a-H2T | 1316bp,seqNo38-1364,HongrES2 | EcoRI, Xhol |
| Plenti-H2-2 | 997bp,seqNo592-1588,HongrES2 | BamHI, Xhol |
| Pcmv-tag4a-CES7 | 1978bp seqNo116-2094 CES7 | EcoRI, HindIII |
| PRL-TK-wt(mut) | 271bp, seqNo1858-2129,CES7 | xbaI, NotI |
